# Trimerization of 1,2‐Diaminocyclohexane Catalyzed by a Metal–Organic Cage Tandem Catalyst with Dual Biomimetic Active Sites

**DOI:** 10.1002/advs.202501872

**Published:** 2025-05-08

**Authors:** Xiao‐Jun Chai, Zhu Zhuo, Zi‐Ang Nan, Shiqiang Mu, Zhong‐Yu Peng, Ting Chen, Wei Wang, Li‐Feng Lin, Xi Meng, Mao‐Chun Hong, You‐Gui Huang

**Affiliations:** ^1^ State Key Laboratory of Structure Chemistry Fujian Institute of Research on the Structure of Matter Chinese Academy of Science Fuzhou Fujian 350002 China; ^2^ Fujian Science & Technology Innovation Laboratory for Optoelectronic Information of China Fuzhou Fujian 350108 China; ^3^ Xiamen Key Laboratory of Rare Earth Photoelectric Functional Materials Xiamen Institute of Rare Earth Materials Haixi Institutes Chinese Academy of Sciences Xiamen Fujian 361021 China; ^4^ University of Chinese Academy of Sciences Beijing 100049 China; ^5^ Institute of Next Generation Matter Transformation College of Material Sciences Engineering at Huaqiao University 668 Jimei Blvd Xiamen Fujian 361021 China; ^6^ Institute of Urban Environment Chinese Academy of Sciences Xiamen Fujian 361021 China

**Keywords:** cascade reactions, dual active sites, enzyme‐like, metal–organic cage, tandem catalyst

## Abstract

Cascade reactions that form multiple chemical bonds in one synthetic step are important for the synthesis of complex molecules. Molecular catalysts for cascade reactions generally require two or more catalytic centers, yet anchoring distinct catalytic centers onto a single molecular catalyst remains extremely challenging. Here a metal–organic cage (MOC) [(ClO_4_)_2_@Zn_20_(L)_8_(HCO_2_)_6_(OH)_6_(H_2_O)_8_]^2+^ (**Zn_20_‐MOC**) (H_3_L = tris(2‐benzimidazolylmethyl)amine) carrying dual biomimetic active sites is reported, i.e. mononuclear {Zn^II^(L)(H_2_O)} and dinuclear {L_2_Zn^II^(CHO_2_)(OH)Zn^II^L_2_}. The two active sites play different roles in the catalytic cascade trimerization of 1,2‐diaminocyclohexane **1**), which produces hexadecahydro‐5a,11a‐butanoquinoxalino[2,3‐b]quinoxaline **2**) with a yield of ≈71.32%. It is find that the mononuclear {Zn^II^(L)(H_2_O)} site catalyzes the dimerization reaction of **1**, yielding an intermediate product 1,2,3,4,4a,6,7,8,9,10a‐decahydrophenazine (**inter. 4**). Further reaction between **1** and **inter. 4** to form the final product must be catalyzed by the dinuclear {L_2_Zn^II^(HCO_2_)(OH)Zn^II^L_2_} site. This work not only provides a new approach to designing catalysts for cascade reactions, but also develops a unique synthetic strategy for the trimerization of 1,2‐diaminocyclohexane, a process that has been unexplored until now.

## Graphical abstract

Dual biomimetic active sites, i.e., mononuclear {Zn^II^(L)(H_2_O)} and dinuclear {L_2_Zn^II^(CHO_2_)(OH)Zn^II^L_2_}, have been successfully intergrated into a metal–organic cage, **Zn_20_‐MOC**. These two different active sites in **Zn_20_‐MOC** catalyze different steps of cascade trimerization of 1,2‐diaminocyclohexane. The mononuclear {Zn^II^(L)(H_2_O)} site catalyzes the initial dimerization, while the further merization producing the trimerization product must be catalyzed by the dinuclear {L_2_Zn^II^(HCO_2_)(OH)Zn^II^L_2_} site.

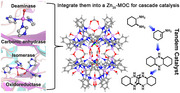

## 1 Introduction

Complex organic molecules, of great importance to pharmaceutical and industrial chemistry, are typically synthesized from small building blocks via multistep reactions.^[^ ^1^, ^2^, ^3^ ^]^ These multistep synthetic procedures are often challenging and with limitations, because of the costly and time‐consuming purification processes required for intermediate products. To overcome these challenges, developing energy‐ and resource‐efficient synthetic methodologies for the one‐pot transformation of simple precursors into complex targeted molecules becomes the holy grail of synthetic organic chemistry.^[^ ^4^, ^5^, ^6^, ^7^, ^8^, ^9^, ^10^ ^]^ In nature, cascade reactions take place on enzymes, and the inter‐enzyme substrate channeling directs the product formation.^[^ ^11^, ^12^ ^]^ However, replicating the sequential catalytic processes found in nature with artificial catalysts has proven to be quite challenging, because it requires precise control over the spatial distribution and connectivity of catalytic species to enable the desired substrate diffusion path.

In this context, the concept of tandem catalysis has emerged. Tandem catalysis is defined as a process in which cascade reactions occur via at least two distinct catalytic mechanisms, with all catalytic species present from the outset of the reaction.^[^ ^13^, ^14^, ^15^, ^16^, ^17^, ^18^, ^19^, ^20^ ^]^ The main challenge in designing tandem catalysts lies in the effective integration of multiple catalytic species into a single catalyst, with each species enabling one particular step of the cascade reaction. To achieve this, common approaches follow two general strategies: i) creating hybrid systems affording the sequestration of individual catalytic species at different parts to prevent their chemical incompatibility;^[^ ^6^, ^21^, ^22^, ^23^, ^24^, ^25^ ^]^ ii) rational design of metal complexes with multiple catalytic centers to catalyze orthogonal cascade reactions (i.e., subsequent reactions can only occur after the initial step).^[^ ^26^, ^27^, ^28^, ^29^, ^30^ ^]^ As an example of the latter, multiple catalytic centers have been integrated into coordination polymers either by in situ synthesis^[^ ^27^ ^]^ or by post‐synthetic^[^ ^26^ ^]^ modifications to produce heterogeneous catalysts. But so far, discrete molecular species with multiple catalytic sites that are capable of homogeneously catalyzing cascade reactions have been relatively less addressed.

As a great example of a natural catalyst, zinc enzymes with Zn^2+^ ions as the active sites play important roles in biological systems. They exist in almost all fundamental enzyme families, including oxidoreductases, transferases, hydrolases, lyases, isomerases, and ligases.^[^ ^31^, ^32^, ^33^, ^34^, ^35^, ^36^ ^]^ Inspired by the versatility of zinc enzymes, synthetic chemists have made great efforts to mimic their structures and functionalities.^[^ ^37^, ^38^, ^39^ ^]^ Among the various active sites in zinc enzymes, the mononuclear tetrahedral {(His)_3_Zn^II^‐OH_2_} site and the dinuclear {(His)_2_Zn^II^(RCO_2_)(OH)Zn^II^(His)_2_} site represent the two most common structural motifs.^[^ ^31^ ^]^ In {(His)_3_Zn^II^‐OH_2_}, Zn(II) is coordinated by three histidine (His) residues and one catalytically important H_2_O ligand. In the dinuclear {(His)_2_Zn^II^(RCO_2_)(OH)Zn^II^(His)_2_} site, the two zinc ions are bridged by OH^‒^ bridges and carboxylate residues. Synthetic analogs of these two motifs have been widely investigated, some of them exhibiting promising biomimetic catalytic properties.^[^ ^40^, ^41^ ^]^ Based on these reports, we envisage that the integration of both motifs into a single molecule may lead to a bifunctional catalyst, capable of catalyzing cascade reactions. However, in spite of tremendous efforts,^[^ ^42^, ^43^, ^44^ ^]^ these two biomimetic sites have not been successfully anchored onto the same molecule, let alone the investigations of their synergetic catalysis.

In this work, biomimetic mononuclear {Zn^II^L(H_2_O)} and dinuclear {L_2_Zn^II^(HCO_2_)(OH)Zn^II^L_2_} sites are successfully integrated into a metal‐organic cage, **Zn_20_‐MOC**. The **Zn_20_‐MOC** catalyzes the trimerization reaction of **1** effectively, forming **2**, which may potentially serve as a DNA strand‐breakage reagent.^[^ ^45^ ^]^ The reported synthetic method for **2** involves the addition of 1,2‐cyclohexanedione and *cis/trans*‐1,2‐cyclohexanediamine.^[^ ^45^ ^]^ We investigate the catalytic reaction process in detail, and propose a reaction mechanism involving the two biomimetic zinc sites. These results may provide important insights into the rational design of tandem catalysts for cascade reactions.

## 2 Results and Discussion

### 2.1 Synthesis and Structural Characterization of Zn_20_‐MOC

The most difficult part of creating a tandem catalyst is how to integrate multiple active sites onto one molecule. By carefully analyzing the structure of the zinc enzyme, we find that the coordination between Zn(II) and the imidazole groups from His residues exists as common structural features in the previously mentioned two active sites. In our previous work, we have studied the self‐assembly of coordination molecule {M^II^H_3_LX} (M = metal ion, H_3_L = tris(2‐benzimidazolylmethyl)amine, X = terminal ligand), driven by anion coordination and *π*···*π* interaction, and have isolated a series of *π*‐stack polyhedra.^[^ ^46^, ^47^, ^48^ ^]^ In all of these self‐assemblies, the L ligands in the {M^II^H_3_LX} molecule are not deprotonated, with the three exterior N–H donors coordinating to various anions. Upon deprotonation, {M^II^LX} may coordinate to metal ions instead of anions, potentially forming metal‐organic cages (MOCs) with dual metal active sites.

Keeping this idea in mind, we conduct a solvothermal reaction of Zn(ClO_4_)_2_·6H_2_O and H_3_L in DMF at 140 °C and obtain single crystals of [(ClO_4_)_2_@Zn_20_(L)_8_(HCO_2_)_6_(OH)_6_(H_2_O)_8_](Cl)_2_·16DMF·H_2_O (**Zn_20_‐MOC‐SC**). HCO_2_^‒^ is generated from the hydrolysis of DMF,^[^ ^49^ ^]^ while Cl^‒^ is generated from the reduction of ClO_4_^‒^,^[^ ^50^ ^]^ which is confirmed by analyzing the reaction solution with ion spectroscopy (Figure S1, Supporting Information). [(ClO_4_)_2_@Zn_20_(L)_8_(CHO_2_)_6_(OH)_6_]^2+^ can be identified from the DMF solution of **Zn_20_‐MOC‐SC** by HR‐ESI‐MS (Figure S2, Supporting Information), suggesting the formation of a self‐assembled MOC consisting of twenty Zn^2+^ ions and eight L ligands. Furthermore, diffusion ordered ^1^H NMR (DOSY) also indicated the formation of **Zn_20_‐MOC**, with a diffusion band at the diffusion coefficient D = 0.9 × 10^‒10^ m^2^ s^‒1^, corresponding to the diameter of ≈ 2.4 nm (Figure S3, Supporting Information).

**Figure 1:**
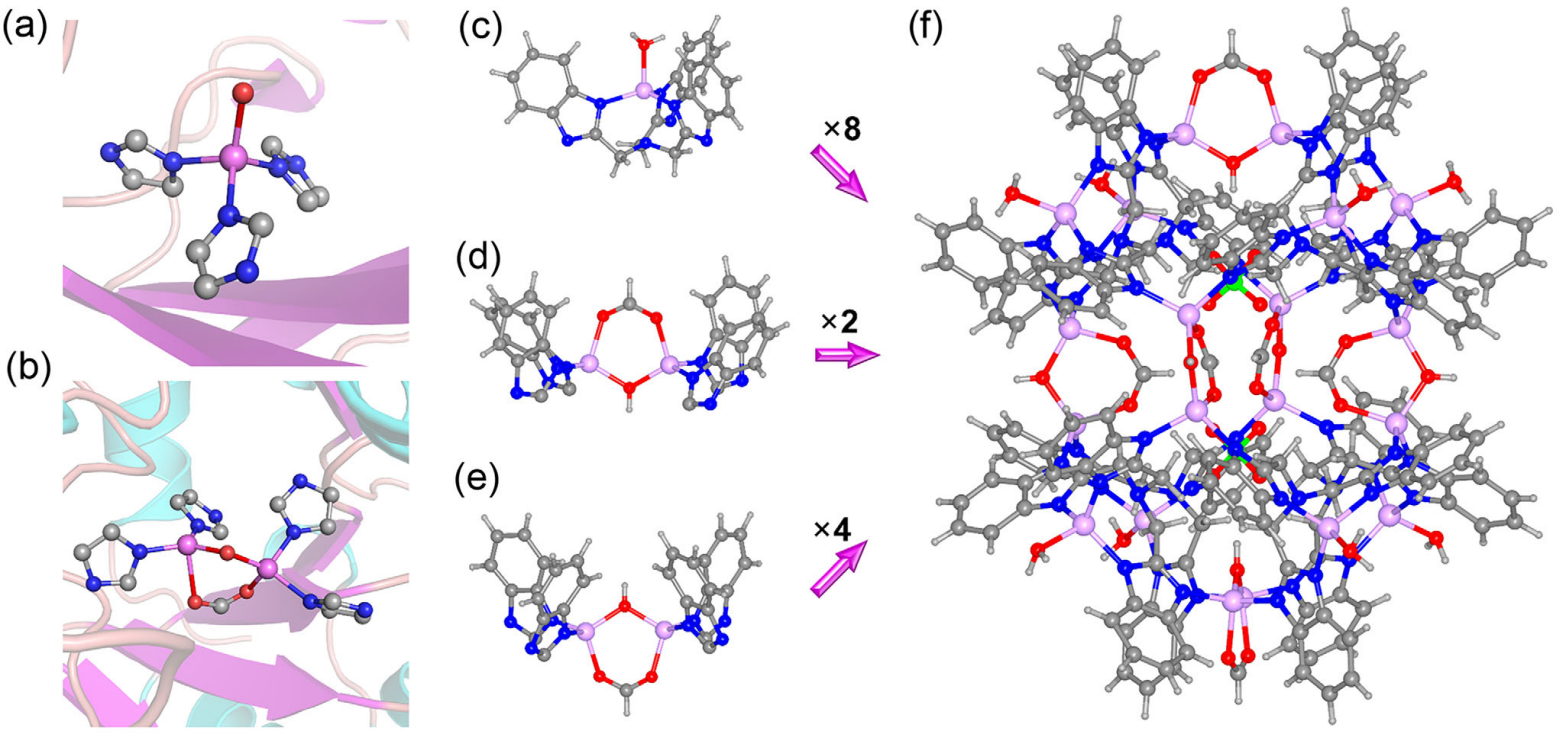
a) The mononuclear {(His)_3_Zn^II^‐H_2_O} active center in zinc enzymes. b) The dinuclear {(His)_2_Zn^II^(RCO_2_)(OH)Zn^II^(His)_2_} active center in zinc enzymes. c) The mononuclear {Zn^II^L(H_2_O)} active center in **Zn_20_‐MOC**. The dinuclear {L_2_Zn^II^(HCO_2_)(OH)Zn^II^L_2_} active center with the *μ*‐OH group pointing outward d) and inward e) to the center of the cage. f) Crystal structure of **Zn_20_‐MOC**.

Single‐crystal X‐ray diffraction study on the **Zn_20_‐MOC‐SC** reveals the formation of a hexahedral cage. **Zn_20_‐MOC‐SC** crystallizes in the cubic *F*‒43*c* space group with the lattice parameter *a* = 66.5279 Å (Table S1, Supporting Information). **Zn_20_‐MOC** is prolate‐shaped with *S*_4_ symmetry and can be best described as a hexahedron with a diameter of ∼2.3 nm, consistent with the ^1^H NMR (DOSY) result (**Figure** **1**f; Figure S3, Supporting Information). The twenty Zn^2+^ ions in **Zn_20_‐MOC** can be classified into two groups. The Zn^2+^ ions on the vertexes are coordinated by one L ligand and one H_2_O ligand, forming a mononuclear {Zn^II^(L)(H_2_O)} motif akin to the {(His)_3_Zn^II^‐H_2_O} site in zinc enzymes (Figure 1a). The other Zn^2+^ ions form the dinuclear {L_2_Zn^II^(HCO_2_)(OH)Zn^II^L_2_} motifs, similar to the {(His)_2_Zn^II^(RCO_2_)(OH)Zn^II^(His)_2_} site in zinc enzymes (Figure 1b). Vertexes of the hexahedron are all occupied by mononuclear {Zn^II^(L)(H_2_O)} motifs (Figure 1c), which are connected by six dinuclear {L_2_Zn^II^(HCO_2_)(OH)Zn^II^L_2_} motifs locating on the facets of **Zn_20_‐MOC** hexahedron. The geometry of **Zn_20_‐MOC** is similar to a 14‐nuclear Co^II^ hexahedron showing photocatalytic activity for producing H_2_O_2_ in pure water.^[^ ^51^ ^]^ The HCOO^‒^ anions in the axial direction (Figure 1d) point outward, while those on the equatorial plane (Figure 1e) point inward to the center of the hexahedron. All benzimidazoylmethyl arms of the eight L ligands also point outward, forming twelve fused calixarene‐like cups. These calixarene‐like cups locate on the edges of the hexahedron, and each of them accommodates a DMF molecule (Figure S4a, Supporting Information). These calixarene‐like cups can be categorized into three groups, i.e., {Zn_2_(ZnH_2_O)_2_(arm)_4_} (I), {[Zn_2_(HCOO)(OH)](ZnH_2_O)_2_Zn(arm)_4_} (II), and {[Zn_2_(HCOO)(OH)]_2_(ZnH_2_O)_2_(arm)_4_} (III) (arm = benzimidazoylmethyl group) (Figure S4b, Supporting Information), leading to three groups of edges with different length (each group contains four equivalent edges). The hexahedral cage is porous with the inner cavity occupied by two ClO_4_^‒^ anions in close proximity^[^ ^52^ ^]^ (Figure 1f; Figure S5, Supporting Information), which are captured by CH···O and OH···O hydrogen bonding. In the lattice of **Zn_20_‐MOC‐SC**, each **Zn_20_‐MOC** associates with its eight neighbors through the synergistic effect of hydrogen bonding and amide‒*π* interactions, forming a supramolecular framework with ***reo*** topology (Figure S6, Supporting Information). However, the framework structure of **Zn_20_‐MOC‐SC** is unstable after desolvation at 90 °C for 6 h as indicated by powder X‐ray diffraction (PXRD) and Brunauer‒Emmett‒Teller (BET) surface area measurements (Figure S7, Supporting Information).

### 2.2 Trimerization of 1,2‐Diaminocyclohexane Catalyzed by Zn_20_‐MOC

Since the two biomimetic catalytic motifs, i.e., the mononuclear {Zn^II^(L)(H_2_O)} and dinuclear {L_2_Zn^II^(CHO_2_)(OH)Zn^II^L_2_}, have been successfully integrated into a single metal–organic cage **Zn_20_‐MOC**, we proceed to investigate the catalytic properties. We focus on the catalytic trimerization of 1,2‐diaminocyclohexane (**1**) to form hexadecahydro‐5a,11a‐butanoquinoxalino[2,3‐b]quinoxaline (**2**), which is a fused heterocycle with potential pharmacological activity on DNA strand‐breakage.^[^ ^45^ ^]^ The synthetic procedure involves cascade reactions of **1**. Therefore, tandem catalysis exploiting both {Zn^II^(L)(H_2_O)} and {L_2_Zn^II^(CHO_2_)(OH)Zn^II^L_2_} centers may be achievable in **Zn_20_‐MOC**.

**Scheme 1:**
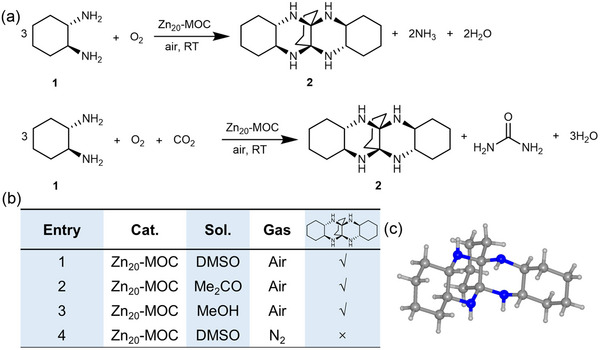
a) The scheme for the trimerization of 1,2‐diaminocyclohexane with (up) and without (down) CO_2_ participation. b) Entries for the trimerization of 1,2‐diaminocyclohexane in various solvents. c) Molecular structure of **2**.

The phase purity for **Zn_20_‐MOC‐SC** powder has been confirmed by powder X‐ray diffraction (PXRD) (Figure S7a, Supporting Information). Using **Zn_20_‐MOC** as the catalyst, the substrate racemic 1,2‐diaminocyclohexane (**1**) readily forms hexadecahydro‐5a,11a‐butanoquinoxalino[2,3‐b]quinoxaline (**2**) in dimethyl sulfoxide (DMSO) under ambient condition (Entry. 1). The yield is determined to be ≈71.32% using High‐Performance Liquid Chromatography Mass Spectrometry (HPLC‐MS) method, giving a turnover number (TON) of 378 (Tables S8 and S9 and Figure S8, Supporting Information). This result clearly demonstrates **Zn_20_‐MOC** as an effective catalyst, although the catalyst cannot be recycled which is an issue for a lot of homogeneous catalysts.^[^ ^53^, ^54^ ^]^ The reaction pathway from **1** to **2** involves deamination and the byproduct NH_4_^+^ is confirmed by Nessler's reagent (Figure S9, Supporting Information). Meanwhile, urea is identified from the resulting solution by electrospray ionization mass spectrometry (ESI‐MS) (Figure S10, Supporting Information). These results indicate that the deamination occurs via two possible pathways, i.e., with (**Scheme** **1**a up) or without (Scheme 1a down) the participation of CO_2_. In the air, the catalytic trimerization proceeds in various solvents, including DMSO (Entry. 1), acetone (Entry. 2), and CH_3_OH (Entry. 3). Due to the poor solubility of **Zn_20_‐MOC‐SC** in acetone and CH_3_OH, the yields and TONs for Entry. 2 and 3 were not determined. However, the reaction is hindered in N_2_ atmosphere (Entry. 4) (Scheme 1b and Figures S11–S14, Supporting Information). This indicates that dehydrogenation, as a necessary step of the trimerization reaction, is induced by O_2_ in air.

Keeping the resulting solution undisturbed for solvent evaporation, single crystals of **2** can be readily isolated. The ^1^H NMR spectrum for **2** is shown in Figure S15 (Supporting Information). Single crystal X‐ray analysis (SCXRD) reveals that **2** crystallizes in *P*2_1_/*c* as a racemic mixture (Scheme 1c, Figure S16, and Table S2, Supporting Information). For both enantiomers, the two 1,2‐di‐iminocyclohexane substituents remain in the *trans‐*conformation, while all of the four chiral carbon centers in the same molecule exhibit the same handedness. The H atoms from the imine groups, identified by Fourier electron density difference maps, are located at both the *cis*‐ and *trans*‐positions (Figure S17, Supporting Information). **2** in the same configuration is also observed in a co‐crystal of **1** and **2** (**2_2_·1**) (Table S3 and Figure S18, Supporting Information), indicating the observed configuration is probably the most stable.

### 2.3 Identification of the Enzyme‐Like Catalytic Sites in Zn_20_‐MOC

**Scheme 2:**
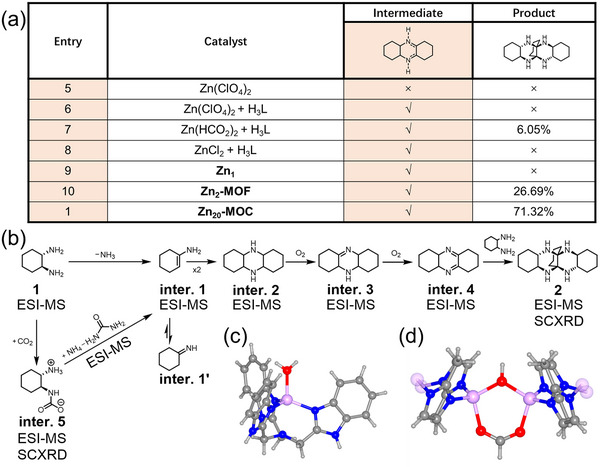
a) Control experiments with different reagents as catalysts. b) The proposed procedure for trimerization of 1,2‐diaminocyclohexane. c) The enzyme‐like {Zn^II^(L)(H_2_O)} site in **Zn_1_**_._ d) The enzyme‐like dinuclear {Zn^II^(CHO_2_)(OH) Zn^II^} site in **Zn_2_‐MOF**.

To provide insights into the catalytic mechanism of **Zn_20_‐MOC**, we perform a series of control experiments (Entries. 5‒8) (**Scheme** **2**a). **2** cannot be produced except for Entry. 7 with a mixture of Zn(HCOO)_2_ and H_3_L as the catalyst (yield of **2**: 6.05%). It is noteworthy that the key intermediates of the trimerization process tetradecahydrophenazine (**inter. 2**) and/or its corresponding dehydrogenation products (i.e. 1,2,3,4,4a,5,5a,6,7,8,9,9a‐dodecahydrophenazine (**inter. 3**) and 1,2,3,4,4a,6,7,8,9,10a‐decahydrophenazine (**inter. 4**)) can be identified by ESI‐MS from the reaction solution of Entry. 6‒8 (Figures S19–S22, Supporting Information). These results suggest that the trimerization occurs via two cascade steps, and the whole trimerization procedure is proposed in Scheme 2b. The initial step involving the deamination and the subsequent dimerization and oxidation produces **inter. 4**. This step can be catalyzed by both the mononuclear {Zn^II^(L)(H_2_O)} and dinuclear {L_2_Zn^II^(CHO_2_)(OH)Zn^II^L_2_} sites. However, the subsequent addition reaction between **1** and **inter. 4** yielding the final product **2** must be catalyzed by the dinuclear site.

To verify our hypothesis, we synthesize two other complexes, [ZnL(H_2_O)]·2(ClO_4_)·solvent (**Zn_1_**) and {[Zn_2_(HCO_2_)(OH)(MeIm)_2_]·DMSO}_n_ (MeIm = deprotonated 2‐methy‐imidazole) (**Zn_2_‐MOF**), each with one enzyme‐like active site. Both structures are characterized by SCXRD (Tables S4 and S5, Supporting Information). **Zn_1_** is a mononuclear complex with the same enzyme‐like mononuclear {Zn^II^(L)(H_2_O)} site as the one in **Zn_20_‐MOC** (Scheme 2c and Figure S23, Supporting Information). **Zn_2_‐MOF** is a 2D coordination polymer with the enzyme‐like dinuclear {Zn^II^(CHO_2_)(OH)Zn^II^} site (Scheme 2d and Figure S24, Supporting Information) similar to the dinuclear active site in **Zn_20_‐MOC**. With **Zn_1_** as a homogeneous catalyst (Entry. 9), **inter. 3** and **inter. 4** can be identified by ESI‐MS while **2** is undetected (Figure S25, Supporting Information). With **Zn_2_‐MOF** as a heterogeneous catalyst (Entry. 10), all of the dimerization intermediates (**inter. 2, inter. 3,** and **inter. 4**) can be identified with the trimerization product **2** (yield for **2**: 26.69%) (Figure S26, Supporting Information). The relatively low yield is probably attributed to that **Zn_2_‐MOF** is insoluble in DMSO and thus works as a heterogeneous catalyst. These results are consistent with the above control experiments (Entries. 5‒8), further demonstrating that the initial dimerization can be catalyzed by both the mononuclear and dinuclear sites while the subsequent addition to produce **2** must be catalyzed by the dinuclear site.

### 2.4 Proposed Catalytic Mechanisms

**Figure 2:**
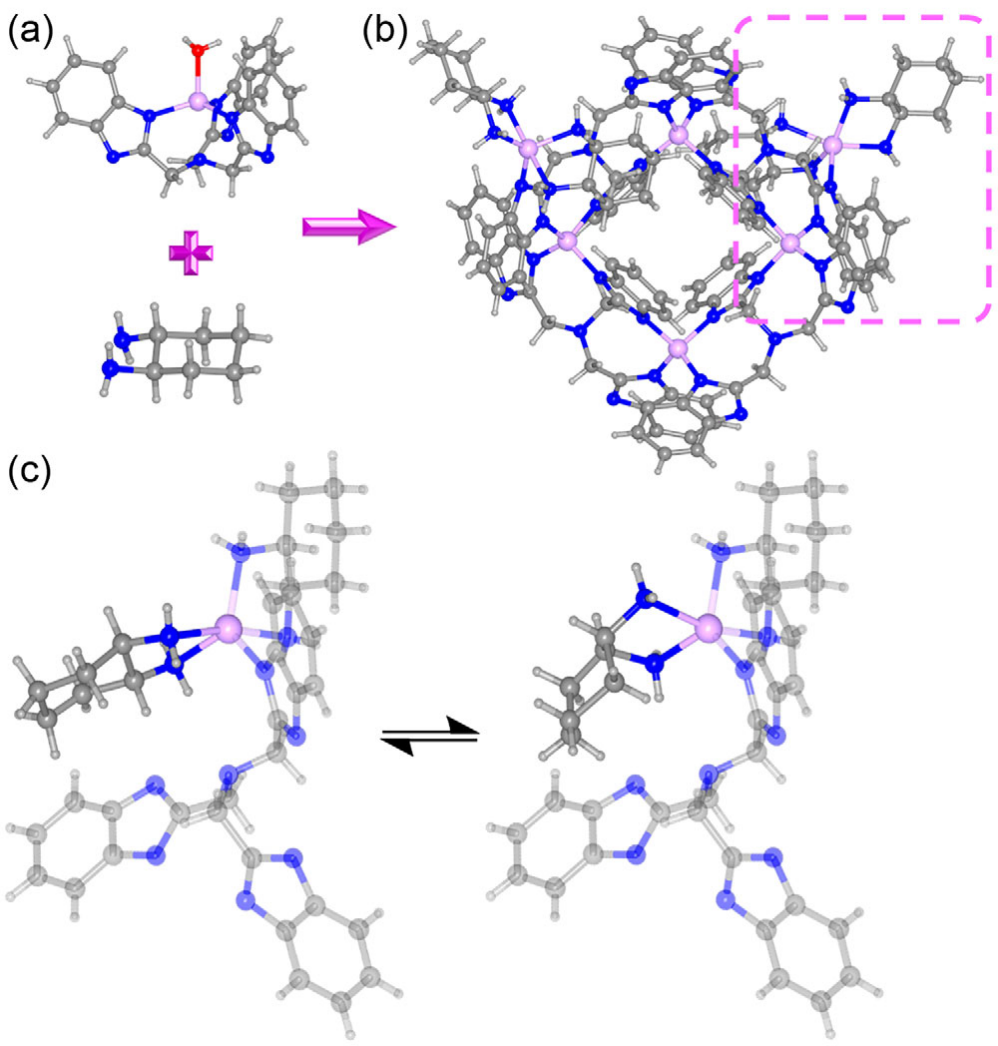
**Zn_1_** reacts with **1** a) giving rise to Zn_6_ b). c) Reversible interconversion between *cis*‐ and *trans*‐**1** occurs at the dangling Zn^2+^ site in **Zn_6_**.

Adding enantiopure *trans*‐(1*R*,2*R*)‐cyclohexane‐1,2‐diamine into the solution of **Zn_1_** in DMSO leads to a yellow solution, from which colorless single crystals of [Zn_6_L_4_(**1**)_4_] (**Zn_6_**) can be isolated (Figure S27, Supporting Information). **Zn_6_** crystallizes in the *P*2_1_ space group (Table S6, Supporting Information). Its asymmetric unit contains two crystallographically independent hexameric {Zn_6_L_4_(**1**)_4_} clusters. This hexameric cluster consists of a saddle‐like {Zn_4_L_4_} square with two {Zn(**1**)_2_} moieties dangling at the diagonal positions (**Figure** **2**a,b). The four Zn^2+^ ions in the {Zn_4_L_4_} square are tetrahedrally coordinated by four N atoms from two L ligands, while the two dangling {ZnL(**1**)_2_} motifs contain Zn^2+^ ions in a trigonal bipyramidal geometry with five N atoms from one L and two **1** ligands. The Zn^II^‒N bond lengths at the dangling {ZnL(**1**)_2_} motifs (2.036‒2.244 Å) are slightly longer than those at the {Zn_4_L_4_} square (1.978‒2.035 Å), implying the lability of the dangling motifs. On one of the two clusters in the asymmetric unit, a disorder of *cis*‐**1** (57%) and *trans*‐**1** (43%) can be observed on the dangling Zn^2+^ ion. This result clearly demonstrates that interconversion between *cis*‐ and *trans*‐**1** occurs at the dangling Zn^2+^ site (Figure 2c).

**Scheme 3:**
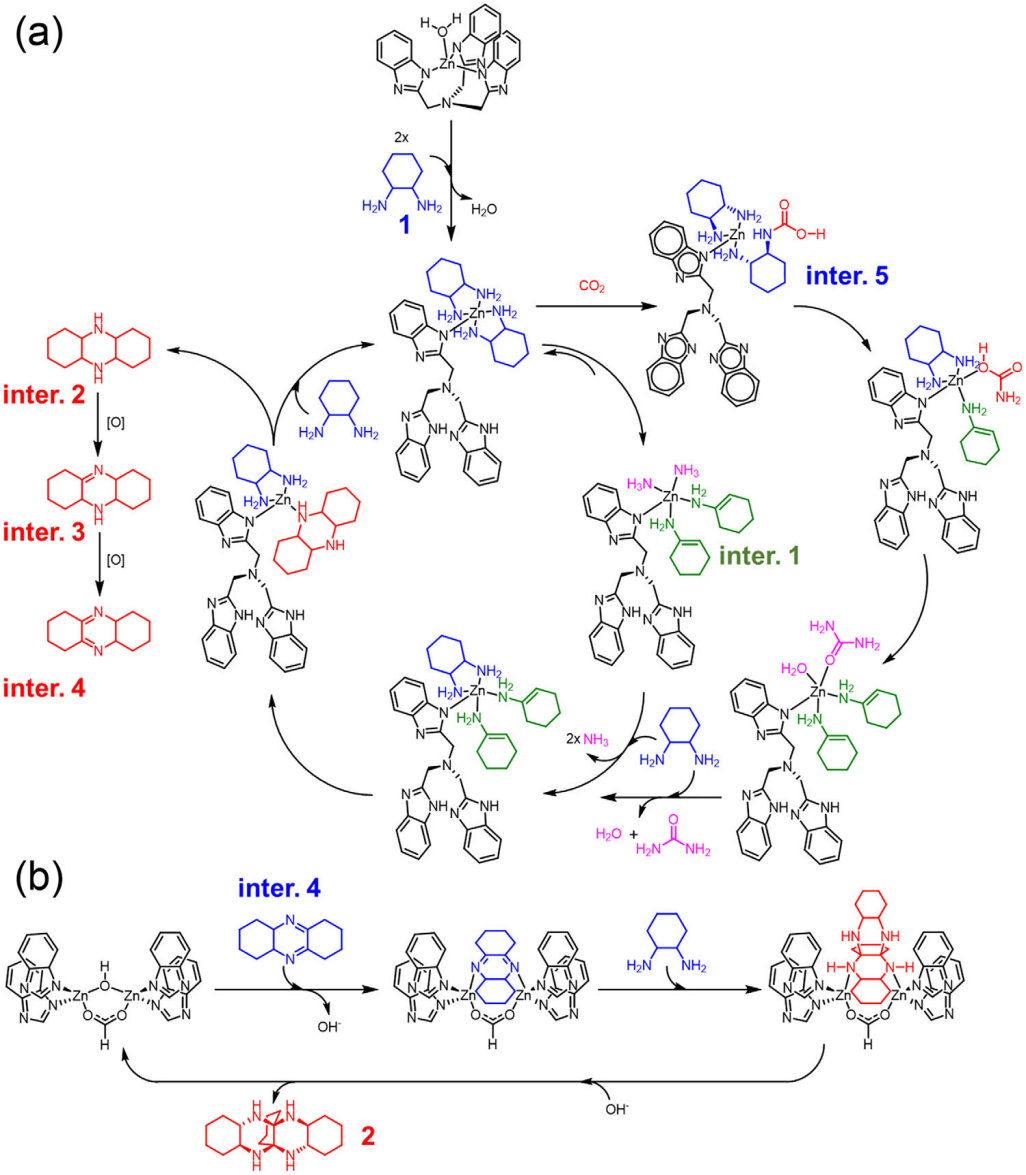
a) Proposed pathways for dimerization of **1** occurred on the mononuclear site. b) Proposed mechanism for the addition reaction between **1** and **inter. 4**.

Based on these observations, it can be envisaged that the chelating coordination between Zn^II^ and L is labile enabling substrate **1** to attack the mononuclear {Zn^II^(L)(H_2_O)} site forming {ZnL(**1**)_2_} to initiate the catalytic reaction. Thus, we propose that the mononuclear {Zn^II^(L)(H_2_O)} site becomes {ZnL(**1**)_2_} under the attack by **1**, followed by the deamination and dimerization reactions. As illustrated in **Scheme** **3**a, after the mononuclear site {Zn^II^(L)(H_2_O)} dissociates from the H_2_O ligand and the L ligand, it coordinates with two molecules of **1,** forming the active site {ZnL(**1**)_2_}. The two coordinated **1** molecules approach each other during this process. Mono‐deamination of **1** occurs spontaneously, affording cyclohex‐1‐en‐1‐amine (**inter. 1**). Next, two **inter. 1** molecule on the same Zn^2+^ site readily dimerized via a [3+3] cyclization addition involving double C‒N couplings, leading to the formation of tetradecahydrophenazine (**inter. 2**). Finally, **inter. 2** was replaced by two molecules of **1** to complete the catalytic cycle. It is worth noting that the dehydrogenation of **inter. 2** spontaneously occurs in air, producing **inter. 3** and **4**.^[^ ^55^ ^]^ Because ligand exchange occurs extremely fast on Zn^2+^,^[^ ^56^ ^]^ **inter. 2, inter. 3, and inter.4** can all be detected in the solution by ESI‐MS.

On the other hand, the deamination of **1** can also occur via an alternative pathway involving CO_2_ insertion.^[^ ^57^ ^]^ We've isolated single crystals of (2‐aminocyclohexyl)carbamic acid (**inter. 5**) from the reaction solution (Table S7 and Figure S28, Supporting Information). This indicates that, at the {ZnL(**1**)_2_} site, the addition reaction between CO_2_ and **1** occurs while coupling with the subsequent proton transfer from the amino group to the carboxylic group, resulting in **inter. 5** (Scheme 3a). The carbamic acid group on **inter. 5** dissociates and then undergoes condensation with one of the amino groups on the other molecule of **1,** leading to 1‐(2‐aminocyclohexyl)urea. The urea group dissociates and can be readily detected by ESI‐MS (Figure S10, Supporting Information). The resulting two **inter. 1** molecules dimerize as described in the first pathway. Regarding the dimerization reaction catalyzed by the dinuclear {L_2_Zn^II^(CHO_2_)(OH)Zn^II^L_2_} site, we propose that two molecules of **1** compete with the L ligand and the OH^‒^ bridge for binding one of the Zn^2+^ ions, creating a {ZnL(**1**)_2_(HCO_2_)} active center. The dimerization of **1** subsequently occurs following the reaction pathway catalyzed by the mononuclear site (Scheme S1).

Meanwhile, because **Zn_1_** cannot catalyze the trimerization of **1**, the addition reaction between **1** and **inter. 4** to form **2** must occur at the dinuclear {L_2_Zn^II^(CHO_2_)(OH)Zn^II^L_2_} site. As shown in Scheme 3b, **inter. 4** competes with the bridging OH^‒^ for coordinating to one of the Zn^2+^ in the dinuclear unit. Subsequently, the [4+2] addition reaction occurs between the coordinated **inter. 4** and **1**, affording **2**. The catalytic cycle is completed by the dissociation of **2** from the Zn^2+^ site, which allows the OH^‒^ bridge to recover its original position.

## 3 Conclusion

In summary, a metal‐organic cage **Zn_20_‐MOC** with dual biomimetic active sites has been synthesized. Simultaneously possessing the mononuclear {Zn^II^(L)(H_2_O)} site and the dinuclear {Zn^II^_2_(CHO_2_)(OH)} site, **Zn_20_‐MOC** serves as a tandem catalyst, effectively catalyzing the trimerization reaction of 1,2‐diaminocyclohexane to form hexadecahydro‐5a,11a‐butanoquinoxalino[2,3‐b]quinoxaline. Control experiments demonstrate that the initial dimerization could be catalyzed by both catalytic sites, while the subsequent trimerization can only be catalyzed by the dinuclear {Zn^II^_2_(CHO_2_)(OH)} site. With the proposed reaction pathway of 1,2‐diaminocyclohexane trimerization, **Zn_20_‐MOC** represents a rare example of enzyme‐like tandem catalysts for conducting cascade reactions. These results provide new insights into the design of biomimetic tandem catalysts, in particular for the multiple C‒N coupling cascade reactions.

## 4 Experimental Section

### Starting Materials

The ligand tris(2‐benzimidazolylmethyl)amine (H_3_L) was synthesized according to the procedure reported in the literature.^[^ ^58^ ^]^ All the other reagents were commercially obtained and used without further purification.

### Synthetic Methods—Synthesis of **Zn_20_‐MOC‐SC**

Tris(2‐benzimidazolylmethyl)amine (H_3_L) (0.925 g, 2.273 mmol) and Zn(ClO_4_)_2_·6H_2_O (1.850 g, 4.973 mmol) were mixed in 125 mL mixed solvent containing 50 mL DMF and 75 mL EtOH. The mixture was sonicated for 20 min, resulting in a clear solution. The resulting solution was charged in a 300 mL autoclave, and then heated at 140 °C for 48 h. The afforded insoluble species were removed by filtration, and the filtrate was kept undisturbed. Colorless block‐shaped crystals can be obtained after ≈2 weeks (yield: 0.590 g, ≈ 40% based on Zn^2+^).

### Synthetic Methods—Synthesis of **Zn_1_ ([ZnL(H_2_O)]·2(ClO_4_)·5.7H_2_O·0.6MeOH)**

The mixture of Zn(ClO_4_)_2_·6H_2_O (74.5 mg, 0.2 mmol) and tris(2‐benzimidazolylmethyl)amine (H_3_L) (82.6 mg, 0.2 mmol) was dissolved in 15 mL MeOH in a glass via by sonication. The resulting clear solution was kept undisturbed at room temperature. Colorless block‐shaped crystals were obtained in one day (yield: 136.7 mg, ≈ 85% based on H_3_L).

### Synthetic Methods—Synthesis of **Zn_2_‐MOF ([Zn_2_(HCO_2_)(OH)(MeIm)]_n_·DMSO)**

The mixture of Zn(NO_3_)_2_·6H_2_O (396 mg, 1.333 mmol), HCOONa (134 mg 1.861 mmol), and 2‐methylimidazole (129.0 mg, 1.573 mmol) was dissolved in the mixed solvent of 4 mL DMSO and H_2_O (v/v = 3/1) in a glass via by sonication. The resulting solution was sealed and heated at 90 °C for 3 weeks. Colorless block‐shaped crystals were obtained (yield: 115 mg, ≈ 40% based on Zn^2+^).

### Synthetic Methods—Synthesis of **Zn_6_ (Zn_6_(L)_4_(1)_4_)**

The mixture of **Zn_20_‐MOC‐SC** (8 mg, 0.00123 mmol) and (+|‒)‐*trans*‐1,2‐diaminocyclohexane (250.3 mg, 2.196 mmol) was dissolved in the mixed solvent of 2.5 mL DMSO and EtOH (v/v = 3/2) in a glass via by sonication. The resulting clear solution was kept undisturbed under ambient conditions. Colorless block‐shaped crystals were obtained after 2 weeks (yield: 3.3 mg, ≈ 80% based on **Zn_20_‐MOC‐SC**).

### Synthetic Methods—Synthesis of Mixed Crystals of Complex **2**, **[1·2_2_]**, and **int.7**

The mixture of **Zn_20_‐MOC‐SC** (8 mg, 0.00123 mmol) and (+|‒)‐*trans*‐1,2‐diaminocyclohexane (250.3 mg 2.196 mmol) was dissolved in 2 mL DMSO by sonication. The resulting clear solution was kept undisturbed under ambient conditions. Mixed colorless block‐shaped crystals of **2, [1·2], and int. 7** were obtained in two weeks. A pure crystalline sample of **2** can be harvested by washing the obtained crystals with the mixed solvents of MeOH and H_2_O (v/v = 4:1).

### Experiments for Different Entries

*Entry 1*: The mixture of **Zn_20_‐MOC‐SC** (9.2 mg, 0.00142 mmol) and (+|‒)‐*trans*‐1,2‐diaminocyclohexane (333.9 mg, 2.929 mmol) was dissolved in 2 mL DMSO by sonication. The resulting clear solution was kept undisturbed under ambient conditions for 4 days, and then 50 µL of the solution was added into 2 mL MeOH for ESI‐MS measurement.

*Entry 2*: The mixture of **Zn_20_‐MOC‐SC** (9.1 mg, 0.00140 mmol) and (+|‒)‐*trans*‐1,2‐diaminocyclohexane (304.8 mg, 2.673 mmol) was dissolved in 2 mL acetone by sonication. The resulting clear solution was kept undisturbed under ambient conditions for 4 days, and then 50 µL of the solution was added into 2 mL MeOH for ESI‐MS measurement.

*Entry 3*: The mixture of **Zn_20_‐MOC‐SC** (9.8 mg, 0.00151 mmol) and (+|‒)‐*trans*‐1,2‐diaminocyclohexane (297.7 mg, 2.611 mmol) was dissolved in 2 mL MeOH by sonication. The resulting clear solution was kept undisturbed under ambient conditions for 4 days, and then 50 µL of the solution was added into 2 mL MeOH for ESI‐MS measurement.

*Entry 4*: In a glove box, the mixture of **Zn_20_‐MOC‐SC** (9.1 mg, 0.00140 mmol) and (+|‒)‐*trans*‐1,2‐diaminocyclohexane (425.5 mg, 3.732 mmol) was dissolved in 2 mL dried DMSO. The resulting clear solution was kept undisturbed in N_2_ atmosphere for four days, and then 50 µL of the solution was added into 2 mL MeOH for ESI‐MS measurement immediately.

*Entry 5*: The mixture of Zn(ClO_4_)_2_·6H_2_O (23.0 mg, 0.0618 mmol) and (+|‒)‐*trans*‐1,2‐diaminocyclohexane (382.1 mg, 3.352 mmol) was dissolved in 2 mL DMSO by sonication. The resulting clear solution was kept undisturbed under ambient conditions for 4 days, and then 50 µL of the solution was added into 2 mL MeOH for ESI‐MS measurement immediately.

*Entry 6*: The mixture of Zn(ClO_4_)_2_·6H_2_O (24.8 mg, 0.0667 mmol), tris(2‐benzimidazolylmethyl)amine (H_3_L) (8.9 mg, 0.0219 mmol), and (+|‒)‐*trans*‐1,2‐diaminocyclohexane (324.2 mg, 2.844 mmol) was dissolved in 2 mL DMSO by sonication. The resulting clear solution was kept undisturbed under ambient conditions for 4 days, and then 50 µL of the solution was added into 2 mL MeOH for ESI‐MS measurement immediately.

*Entry 7*: The mixture of Zn(HCOO)_2_ (18.5 mg, 0.119 mmol), tris(2‐benzimidazolylmethyl)amine (H_3_L) (8.2 mg, 0.0201 mmol), and (+|‒)‐*trans*‐1,2‐diaminocyclohexane (351.8 mg, 3.086 mmol) was dissolved in 2 mL DMSO by sonication. The resulting clear solution was kept undisturbed under ambient conditions for 4 days, and then 50 µL of the solution was added into 2 mL MeOH for ESI‐MS measurement immediately.

*Entry 8*: The mixture of ZnCl_2_ (12.8 mg, 0.0941 mmol), tris(2‐benzimidazolylmethyl)amine (H_3_L) (8.6 mg, 0.0211 mmol), and (+|‒)‐*trans*‐1,2‐diaminocyclohexane (382.1 mg, 3.352 mmol) was dissolved in 2 mL DMSO by sonication. The resulting clear solution was kept undisturbed under ambient conditions for 4 days, and then 50 µL of the solution was added into 2 mL MeOH for ESI‐MS measurement immediately.

*Entry 9*: The mixture of **Zn_1_** (8.2 mg, 0.0108 mmol) and (+|‒)‐*trans*‐1,2‐diaminocyclohexane (269.6 mg, 2.365 mmol) was dissolved in 2 mL DMSO by sonication. The resulting clear solution was kept undisturbed under ambient conditions for 4 days, and then 50 µL of the solution was added into 2 mL MeOH for ESI‐MS measurement immediately.

*Entry 10*: The mixture of **Zn_2_‐MOF** (8.8 mg, 0.0203 mmol) and (+|‒)‐*trans*‐1,2‐diaminocyclohexane (307.5 mg, 2.697 mmol) was dissolved in 2 mL DMSO by sonication. The resulting clear solution was kept undisturbed under ambient conditions for 4 days, and then 50 µL of the solution was added into 2 mL MeOH for ESI‐MS measurement immediately.

### Physical Measurement

Electrospray Ionization Mass Spectrometry (ESI‐MS) measurements were performed on an ABI 3200 (ABI3200QTRAP) system. High‐resolution electrospray Ionization Mass Spectrometry (HR‐ESI‐MS) was performed on an Agilent Technologies ESI‐TOF‐MS. ^1^H NMR was performed on Bruker 500 MHz/Avance III. Ion spectroscopy analysis was performed on an instrument of Metrohm 930 Compact IC Flex.

### Crystallography

Single‐crystal X‐ray data for **Zn_20_‐MOC‐SC** was collected on XtaLAB Synergy diffractometer with Cu‐*K*α, and the data for the other single‐crystals were collected on Bruker D8 Venture diffractometer with Mo‐*K*α radiation micro‐focus X‐ray sources. The CrysAlisPro and APEX software were used to collect and reduce the raw data. The structures were solved by ShelXT with intrinsic phasing and refined on *F* ^2^ using full‐matrix least‐squares methods, with ShelXL and Olex^2^ used as graphical user interfaces. Detailed crystallographic data are listed in Tables S1‒S7 (Supporting Informaton).

### Determination of Yield and Turnover Number (TON)

The yields and TONs for entries 9, 12, 13, and 14 were determined using the High‐Performance Liquid Chromatography Mass Spectrometry (HPLC‐MS) method performed on a ZORBAX Eclipse Plus C18 (2.1 × 50 mm, 1.8 µm, Agilent, USA) column at 40 °C. The detective wavelength was set to 280 nm and the flow rate was 0.3 mL min^−1^. The mobile phase A is 0.1% (v/v) formic acid aqueous solution, and the mobile phase B is MeOH. The measurement parameters are listed in Table S8 (Supporting Information). To accurately determine the yields and TONs, the sample of 5.731 mg of high‐purity crystals of the trimerization product **2** was used as the standard sample. In specific, 5.731 mg of high purity **2** was dissolved in the mixture of MeOH and H_2_O (v/v = 4:1) (1 mL), and then the solution (1 mL) was subject to HPLC‐MS analysis. Similarly, the solid product for Entry 1, 7, and 10 was dissolved with the mixture of MeOH and H_2_O (v/v = 4:1) (25 mL), respectively, and then 1 mL of each solution was subject for HPLC‐MS analysis. The results for HPLC‐MS analyses are shown in Figure S8 (Supporting Information), and the determined yields and TONs are listed in Table S9 (Supporting Information).

### Detection of NH_4_^+^ Using Nessler Reagent

The mixture of **Zn_20_‐MOC‐SC** (8.0 mg, 0.00123 mmol) and (+|‒)‐*trans*‐1,2‐diaminocyclohexane (250.3 mg, 2.196 mmol) was dissolved in 2 mL DMSO. The resulting clear solution was kept undisturbed under ambient conditions, colorless crystals were obtained after 2 weeks. The crystals were isolated by filtration, and then 2 mL of Nessler's reagent solution was added to the filtrate. The filtrate immediately turned from colorless to brown, indicating NH_4_^+^ ions exist in the filtrate. Therefore, deamination of (+|‒)‐*trans*‐1,2‐diaminocyclohexane occurred in the reactant solution.

## Conflict of Interest

The authors declare no conflict of interest.

## Supporting information



Supporting Information

Supporting Information

## Data Availability

The X‐ray crystallographic coordinates for structures reported in this article have been deposited at the Cambridge Crystallographic Data Centre (CCDC), under deposition numbers CCDC 2411546‒2411552. These data can be obtained free of charge from The Cambridge Crystallographic Data Centre via www.ccdc.cam.ac.uk/data_request/cif.
