# Supplementary material for: Trimerization of 1,2‐Diaminocyclohexane Catalyzed by a Metal–Organic Cage Tandem Catalyst with Dual Biomimetic Active Sites
Source: Adv Sci (Weinh). 2025 May 8;12(27):2501872. doi: 10.1002/advs.202501872 (PMC12279243; doi:10.1002/advs.202501872)
Supplement: Supplementary file 1 — Supporting Information [file ADVS-12-2501872-s002.docx]

Supporting Information

Trimerization of 1,2-Diaminocyclohexane Catalyzed by a Metal-Organic Cage Tandem Catalyst with Dual Biomimetic Active Sites

Xiao-Jun Chai, Zhu Zhuo, Zi-Ang Nan,* Shiqiang Mu, Zhong-Yu Peng, Ting Chen, Wei Wang, Li-Feng Lin, Xi Meng, Mao-Chun Hong, and You-Gui Huang*

**Table of Contents**

**S1.** Methods

**S1.1** Crystallography

**S1.2** Determination of Yield and Turnover Number (TON)

**S2.** Structure of Zn_20_-MOC

**S3.** Trimerization of 1,2-Diaminocyclohexane Catalyzed by Zn_20_-MOC

**S4.** Identification of the Enzyme-Like Catalytic Sites in Zn_20_-MOC

**S5.** Proposed Catalytic Mechanism

**S1.** Methods

**S1.1** Crystallography

**Table S1.** Crystal data and structure refinement for **Zn_20_-MOC.**

| Identification code | **Zn_20_-MOC** |
| --- | --- |
| CCDC No. | 2411552 |
| Empirical formula | C_240_H_282_Cl_4_N_70_O_56_Zn_20_ |
| Formula weight | 6492.54 |
| Temperature/K | 100.15 |
| Crystal system | cubic |
| Space group | *F*-43*c* |
| *a*/Å | 66.57639(10) |
| *b*/Å | 66.57639(10) |
| *c*/Å | 66.57639(10) |
| *α*/° | 90 |
| *β*/° | 90 |
| *γ*/° | 90 |
| Volume/Å^3^ | 295094.3(13) |
| *Z* | 24 |
| *ρ*_calc_g/cm^3^ | 0.877 |
| *μ*/mm^‑1^ | 1.640 |
| *F*(000) | 79872.0 |
| Radiation | Cu Kα (*λ* = 1.54184) |
| 2*Θ* range for data collection/° | 5.936 to 153.84 |
| Index ranges | -82 ≤ *h* ≤ 82, -60 ≤ *k* ≤ 83, -84 ≤ *l* ≤ 69 |
| Reflections collected | 412341 |
| Independent reflections | 25639 [*R*_int_ = 0.0303, *R*_sigma_ = 0.0102] |
| Data/restraints/parameters | 25639/73/936 |
| Goodness-of-fit on *F*^2^ | 1.028 |
| Final *R* indexes [*I*>=2*σ* (I)] | *R*_1_ = 0.0269, *wR*_2_ = 0.0849 |
| Final *R* indexes [all data] | *R*_1_ = 0.0296, *wR*_2_ = 0.0874 |
| Largest diff. peak/hole / e Å^-3^ | 0.12/-0.10 |
| Flack parameter | 0.4553(17) |

**Table S2.** Crystal data and structure refinement for **2.**

| Identification code | **2** |
| --- | --- |
| CCDC No. | 2411548 |
| Empirical formula | C_18_H_32_N_4_ |
| Formula weight | 304.47 |
| Temperature/K | 200.00 |
| Crystal system | monoclinic |
| Space group | *P*2_1_/*c* |
| *a*/Å | 9.3178(6) |
| *b*/Å | 16.7577(12) |
| *c*/Å | 11.1446(8) |
| *α*/° | 90 |
| *β*/° | 105.347(3) |
| *γ*/° | 90 |
| Volume/Å^3^ | 1678.1(2) |
| *Z* | 4 |
| *ρ*_calc_g/cm^3^ | 1.205 |
| *μ*/mm^‑1^ | 0.073 |
| *F*(000) | 672.0 |
| Radiation | Mo Kα (*λ* = 0.71073) |
| 2*Θ* range for data collection/° | 5.144 to 53.496 |
| Index ranges | -11 ≤ *h* ≤ 11, -21 ≤ *k* ≤ 21, -14 ≤ *l* ≤ 14 |
| Reflections collected | 33244 |
| Independent reflections | 3557 [*R*_int_ = 0.0451, *R*_sigma_ = 0.0216] |
| Data/restraints/parameters | 3557/0/211 |
| Goodness-of-fit on *F*^2^ | 1.059 |
| Final *R* indexes [*I*>=2*σ* (*I*)] | *R*_1_ = 0.0422, *wR*_2_ = 0.1054 |
| Final *R* indexes [all data] | *R*_1_ = 0.0527, *wR*_2_ = 0.1143 |
| Largest diff. peak/hole / e Å^-3^ | 0.30/-0.20 |

**Table S3.** Crystal data and structure refinement for **2_2_·1**.

| Identification code | **2_2_·1** |
| --- | --- |
| CCDC No. | 2411549 |
| Empirical formula | C_21_H_39_N_5_ |
| Formula weight | 361.57 |
| Temperature/K | 250.00 |
| Crystal system | monoclinic |
| Space group | *C*2/*c* |
| *a*/Å | 15.9924(4) |
| *b*/Å | 9.8705(2) |
| *c*/Å | 25.7336(6) |
| *α*/° | 90 |
| *β*/° | 93.3730(10) |
| *γ*/° | 90 |
| Volume/Å^3^ | 4055.09(16) |
| *Z* | 8 |
| *ρ*_calc_g/cm^3^ | 1.184 |
| *μ*/mm^‑1^ | 0.072 |
| *F*(000) | 1600.0 |
| Radiation | Mo Kα (*λ* = 0.71073) |
| 2*Θ* range for data collection/° | 5.058 to 54.294 |
| Index ranges | -20 ≤ h ≤ 20, -12 ≤ k ≤ 12, -32 ≤ l ≤ 32 |
| Reflections collected | 39858 |
| Independent reflections | 4482 [*R*_int_ = 0.0533, *R*_sigma_ = 0.0255] |
| Data/restraints/parameters | 4482/48/295 |
| Goodness-of-fit on *F*^2^ | 1.056 |
| Final *R* indexes [*I*>=2*σ* (*I*)] | *R*_1_ = 0.0527, *wR*_2_ = 0.1244 |
| Final *R* indexes [all data] | *R*_1_ = 0.0755, *wR*_2_ = 0.1412 |
| Largest diff. peak/hole / e Å^-3^ | 0.27/-0.21 |

**Table S4.** Crystal data and structure refinement for **Zn_1_.**

| Identification code | **Zn_1_** |
| --- | --- |
| CCDC No. | 2411550 |
| Empirical formula | C_24.6_H_34.8_Cl_2_N_7_O_14.3_Zn |
| Formula weight | 793.66 |
| Temperature/K | 200.00 |
| Crystal system | triclinic |
| Space group | *P*-1 |
| *a*/Å | 12.0922(19) |
| *b*/Å | 13.306(2) |
| *c*/Å | 14.188(2) |
| *α*/° | 99.183(6) |
| *β*/° | 113.892(5) |
| *γ*/° | 111.163(6) |
| Volume/Å^3^ | 1817.0(5) |
| *Z* | 2 |
| *ρ*_calc_ g/cm^3^ | 1.451 |
| *μ*/mm^‑1^ | 0.894 |
| *F*(000) | 820.0 |
| Radiation | Mo Kα (*λ* = 0.71073) |
| 2*Θ* range for data collection/° | 4.178 to 58.646 |
| Index ranges | -16 ≤ *h* ≤ 16, -18 ≤ *k* ≤ 18, -19 ≤ *l* ≤ 19 |
| Reflections collected | 72644 |
| Independent reflections | 9835 [*R*_int_ = 0.0742, *R*_sigma_ = 0.0408] |
| Data/restraints/parameters | 9835/75/492 |
| Goodness-of-fit on *F*^2^ | 1.091 |
| Final *R* indexes [*I*>=2*σ* (*I*)] | *R*_1_ = 0.0896, *wR*_2_ = 0.2626 |
| Final *R* indexes [all data] | *R*_1_ = 0.1034, *wR*_2_ = 0.2734 |
| Largest diff. peak/hole / e Å^-3^ | 1.50/-1.16 |

**Table S5.** Crystal data and structure refinement for **Zn_2_-MOF.**

| Identification code | **Zn_2_-MOF** |
| --- | --- |
| CCDC No. | 2411546 |
| Empirical formula | C_11_H_18_N_4_O_4_SZn_2_ |
| Formula weight | 433.09 |
| Temperature/K | 200.00 |
| Crystal system | orthorhombic |
| Space group | *Pnma* |
| *a*/Å | 13.5268(12) |
| *b*/Å | 12.6661(11) |
| *c*/Å | 10.1954(9) |
| *α*/° | 90 |
| *β*/° | 90 |
| *γ*/° | 90 |
| Volume/Å^3^ | 1746.8(3) |
| *Z* | 4 |
| *ρ*_calc_g/cm^3^ | 1.647 |
| *μ*/mm^‑1^ | 2.885 |
| *F*(000) | 880.0 |
| Radiation | Mo Kα (*λ* = 0.71073) |
| 2*Θ* range for data collection/° | 5.13 to 54.984 |
| Index ranges | -15 ≤ *h* ≤ 16, -16 ≤ *k* ≤ 13, -13 ≤ *l* ≤ 13 |
| Reflections collected | 5523 |
| Independent reflections | 2041 [*R*_int_ = 0.0264, *R*_sigma_ = 0.0315] |
| Data/restraints/parameters | 2041/4/117 |
| Goodness-of-fit on *F*^2^ | 1.058 |
| Final *R* indexes [*I*>=2*σ* (*I*)] | *R*_1_ = 0.0285, *wR*_2_ = 0.0697 |
| Final *R* indexes [all data] | *R*_1_ = 0.0354, *wR*_2_ = 0.0736 |
| Largest diff. peak/hole / e Å^-3^ | 0.60/-0.38 |

**Table S6.** Crystal data and structure refinement for **Zn_6_.**

| Identification code | **Zn_6_** |
| --- | --- |
| CCDC No. | 2411551 |
| Empirical formula | C_136.5_H_200_N_36_O_19.5_S_8.25_Zn_6_ |
| Formula weight | 3314.03 |
| Temperature/K | 273.15 |
| Crystal system | monoclinic |
| Space group | *P*2_1_ |
| *a*/Å | 20.628(4) |
| *b*/Å | 27.589(6) |
| *c*/Å | 28.685(6) |
| *α*/° | 90 |
| *β*/° | 94.32(3) |
| *γ*/° | 90 |
| Volume/Å^3^ | 16279(6) |
| *Z* | 4 |
| *ρ*_calc_g/cm^3^ | 1.352 |
| *μ*/mm^‑1^ | 1.046 |
| *F*(000) | 6956.0 |
| Radiation | Mo Kα (*λ* = 0.71073) |
| 2*Θ* range for data collection/° | 3.774 to 55.11 |
| Index ranges | -26 ≤ *h* ≤ 26, -34 ≤ *k* ≤ 35, -37 ≤ *l* ≤ 37 |
| Reflections collected | 331276 |
| Independent reflections | 71104 [*R*_int_ = 0.1182, *R*_sigma_ = 0.1026] |
| Data/restraints/parameters | 71104/677/4028 |
| Goodness-of-fit on *F*^2^ | 1.029 |
| Final *R* indexes [*I*>=2*σ* (*I*)] | *R*_1_ = 0.0777, *wR*_2_ = 0.1874 |
| Final *R* indexes [all data] | *R*_1_ = 0.1440, *wR*_2_ = 0.2398 |
| Largest diff. peak/hole / e Å^-3^ | 1.22/-0.77 |
| Flack parameter | 0.024(4) |

**Table S7.** Crystal data and structure refinement for (2-aminocyclohexyl)carbamic acid (**inter. 5**)**.**

| Identification code | **inter. 5** |
| --- | --- |
| CCDC No. | 2411547 |
| Empirical formula | C_14_H_28_N_4_O_4_ |
| Formula weight | 316.40 |
| Temperature/K | 200.00 |
| Crystal system | monoclinic |
| Space group | *P*2_1_ |
| *a*/Å | 12.249(3) |
| *b*/Å | 5.0872(12) |
| *c*/Å | 13.058(3) |
| *α*/° | 90 |
| *β*/° | 91.599(7) |
| *γ*/° | 90 |
| Volume/Å^3^ | 813.4(3) |
| *Z* | 2 |
| *ρ*_calc_g/cm^3^ | 1.292 |
| *μ*/mm^‑1^ | 0.095 |
| *F*(000) | 344.0 |
| Radiation | Mo Kα (*λ* = 0.71073) |
| 2*Θ* range for data collection/° | 4.498 to 54.68 |
| Index ranges | -15 ≤ *h* ≤ 15, -6 ≤ *k* ≤ 6, -16 ≤ *l* ≤ 16 |
| Reflections collected | 11138 |
| Independent reflections | 3322 [*R*_int_ = 0.0880, *R*_sigma_ = 0.0886] |
| Data/restraints/parameters | 3322/2/207 |
| Goodness-of-fit on *F*^2^ | 1.123 |
| Final *R* indexes [*I*>=2*σ* (*I*)] | *R*_1_ = 0.0806, *wR*_2_ = 0.2355 |
| Final *R* indexes [all data] | *R*_1_ = 0.1188, *wR*_2_ = 0.2607 |
| Largest diff. peak/hole / e Å^-3^ | 0.32/-0.35 |
| Flack parameter | -0.6(10) |

**S1.2** Determination of Yield and Turnover Number (TON)

**Table S8.** Parameters for HPLC-MS analyses.

| Time (min) | Flow Rate (ml/min) | Max. Pressure Limit (bar) | Mobile Phase | |
| --- | --- | --- | --- | --- |
|  |  |  | A (%) | B (%) |
| 0.00 | 0.3 | 1300 | 90 | 10 |
| 1.00 | 0.3 | 1300 | 85 | 15 |
| 2.50 | 0.3 | 1300 | 75 | 25 |
| 3.00 | 0.3 | 1300 | 70 | 30 |
| 5.00 | 0.3 | 1300 | 90 | 10 |
| 7.00 | 0.3 | 1300 | 90 | 10 |

**Table S9.** The yields and TONs determined by HPLC-MS.

| Entry | Catalyst (mmol) | **1** (mmol) | Yield (%) | TON |
| --- | --- | --- | --- | --- |
| 1 | **Zn_20_-MOC**(0.00166) | 2.640 | 71.32 | 378*^c^* |
| 7 | H_3_L(0.026) + Zn(HCOO)_2_(0.044) | 1.887 | 6.05 | 0.86*^a^* |
| 10 | Zn_2_-MOF(0.024） | 2.794 | 26.69 | 10*^b^* |

*^a^*The formula of catalyst is Zn(HCOO)_2_. *^b^*The formula of catalyst is Zn_2_(HCO_2_)(OH)(MeIm)_2_·DMSO. *^c^*The formula of catalyst is [(ClO_4_)_2_@Zn_20_(L)_8_(HCO_2_)_6_(OH)_6_(H_2_O)_8_](Cl)_2_·16DMF·H_2_O

**S2. Structure of Zn_20_-MOC**


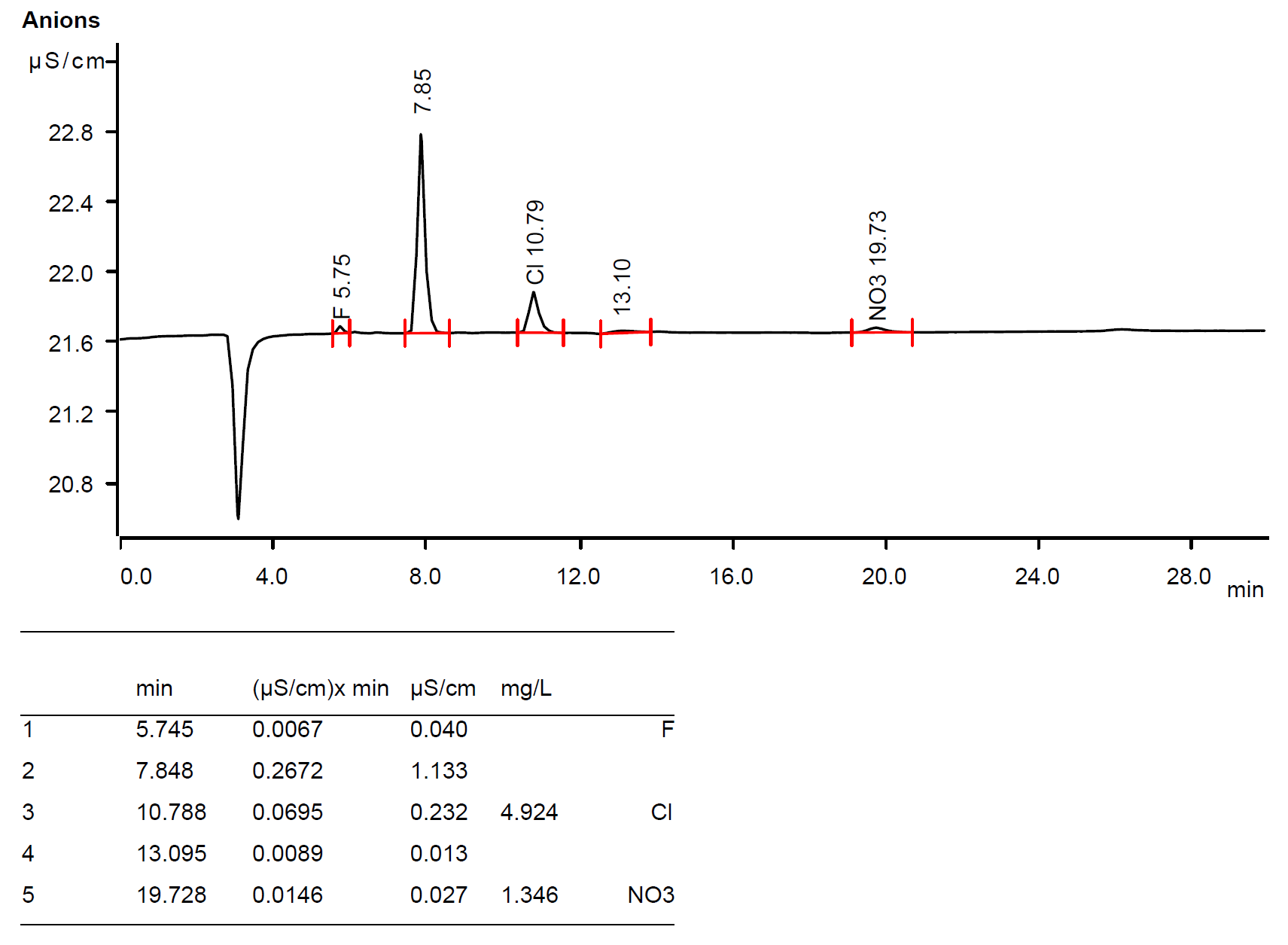


**Figure S1**. The ion spectroscopy of the reactant solution after synthesis of **Zn_20_-MOC**. (Cl^‒^ was detected indicating ClO_4_^‒^ was partially reduced to Cl^‒^)


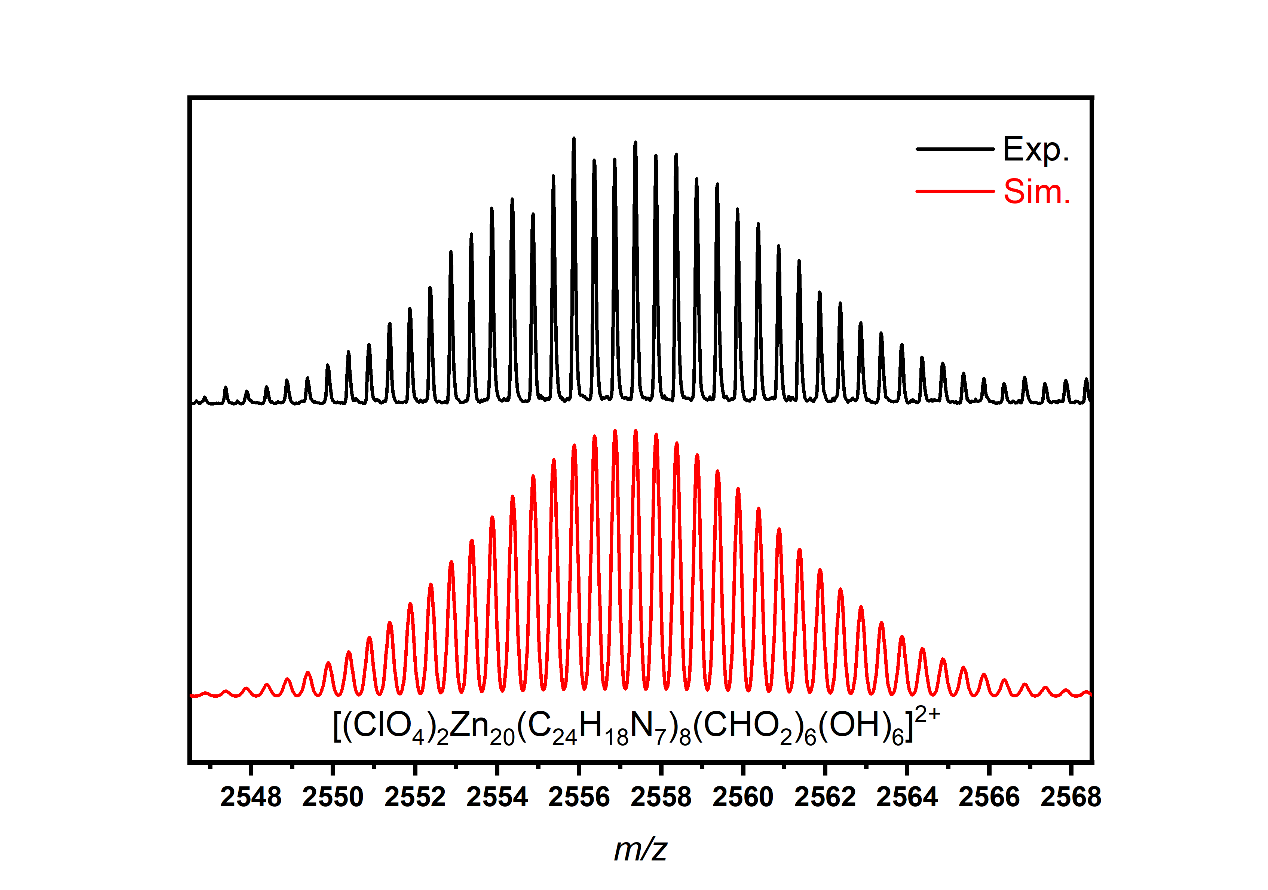


**Figure S2**. HR-ESI-MS for **Zn_20_-MOC**.


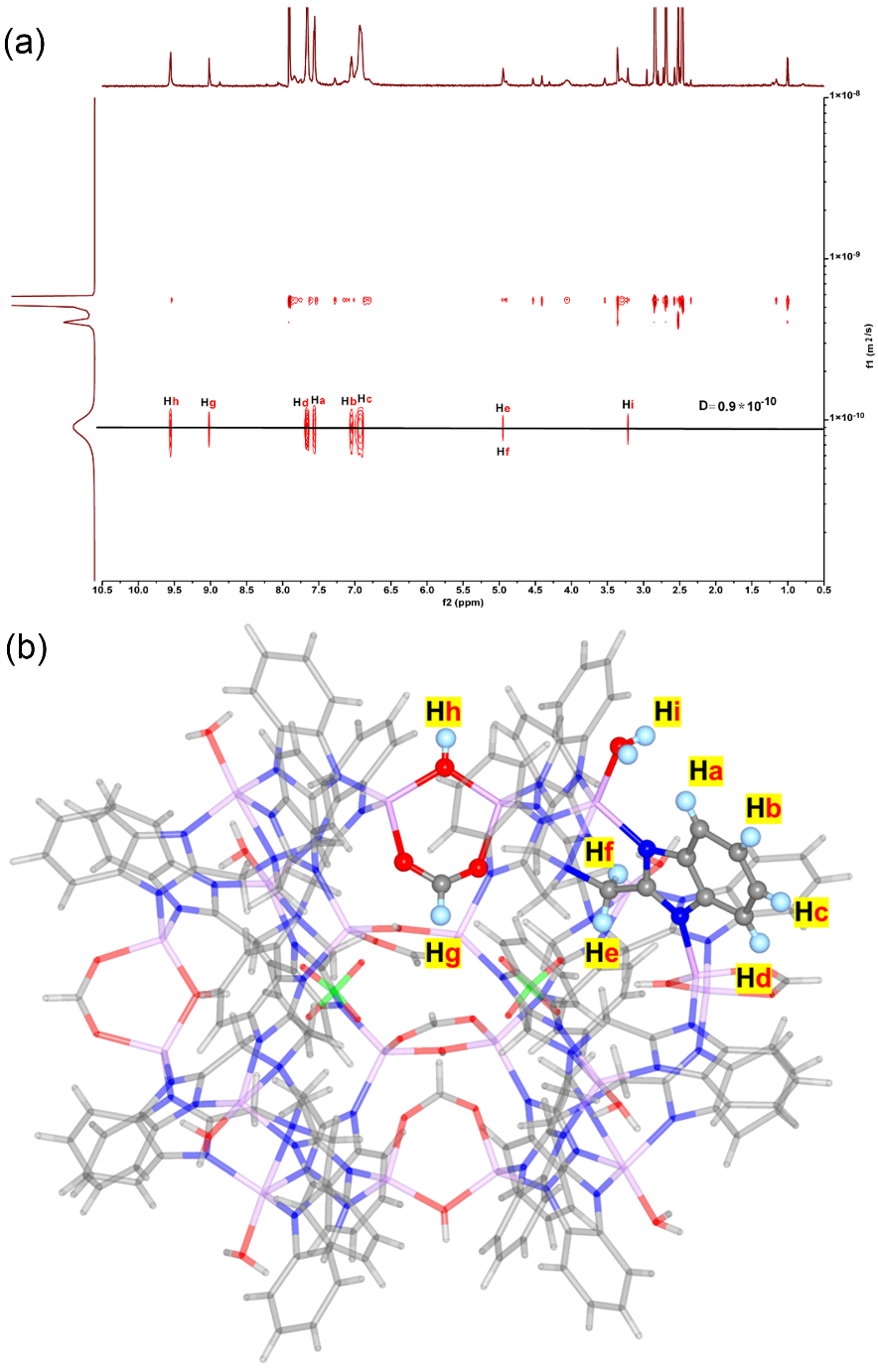


**Figure S3**. ^1^H DOSY for **Zn_20_-MOC-SC** in DMSO.


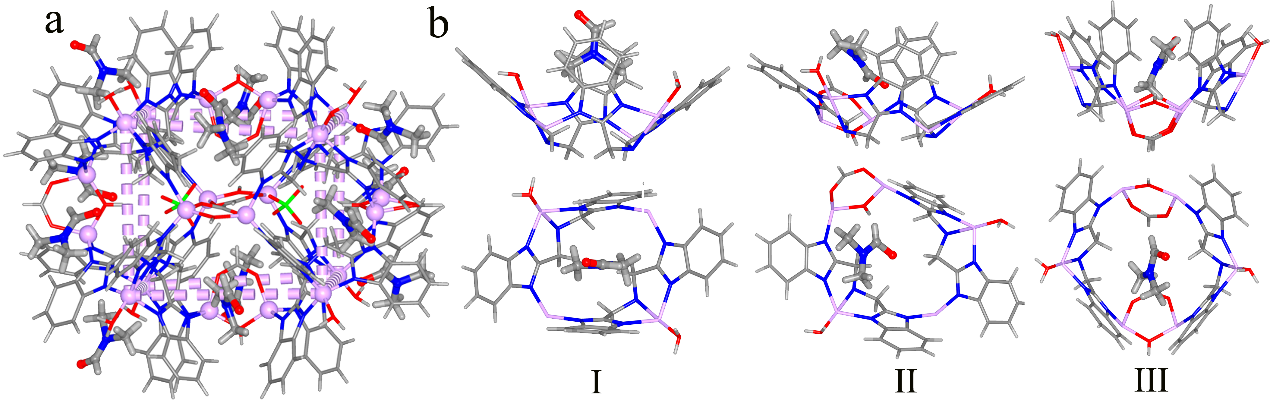


**Figure S4**. Crystal structure of **Zn_20_-MOC**. **a** Structure of **Zn_20_-MOC** showing each calixarene-like cup accommodating a DMF molecule. **b** Side view (up) and top view of the three types of calixarene-like cups.


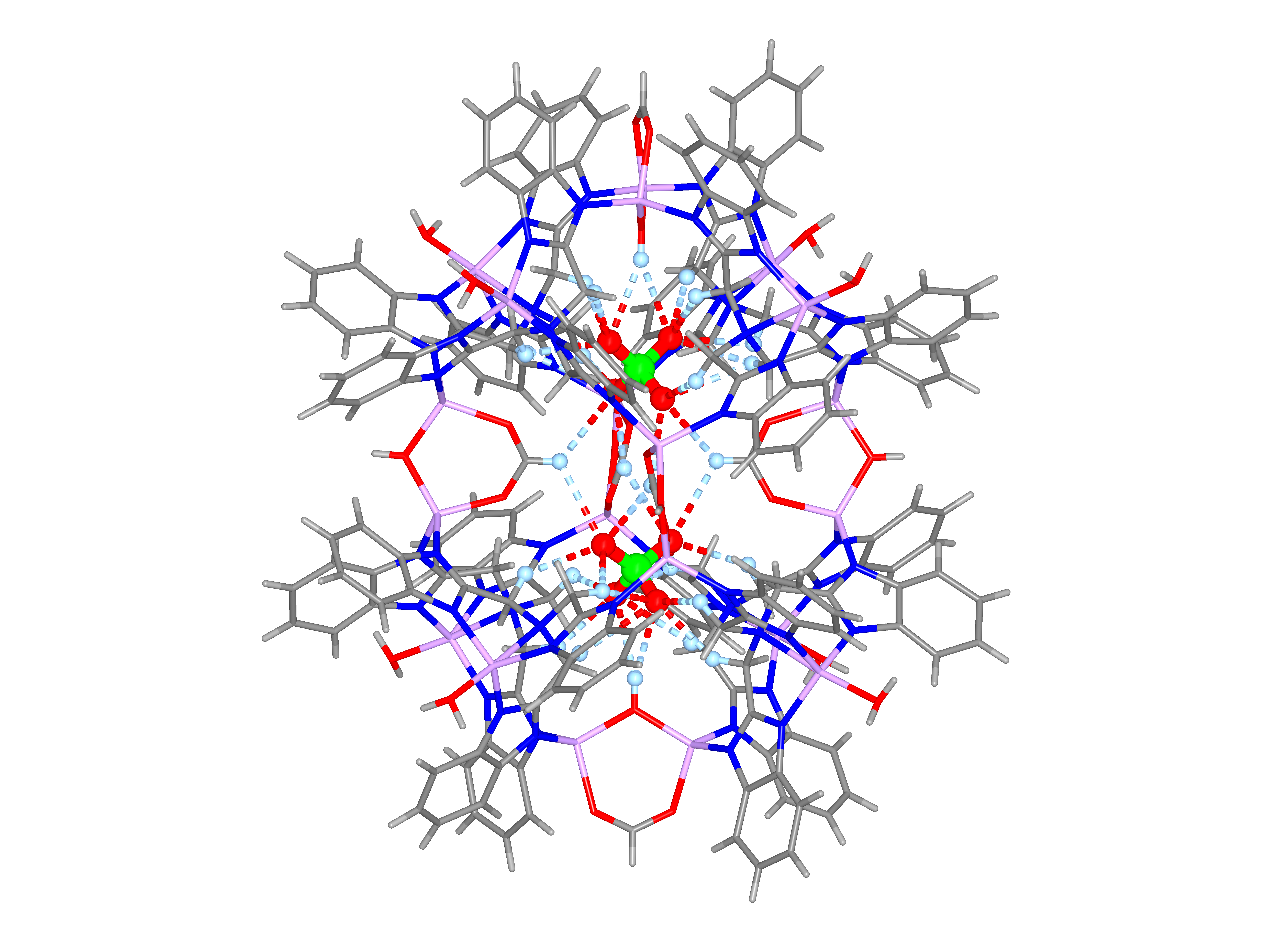


**Figure S5**. **Zn_20_-MOC** capturing two ClO_4_^‒^ anions in the inner cavity.


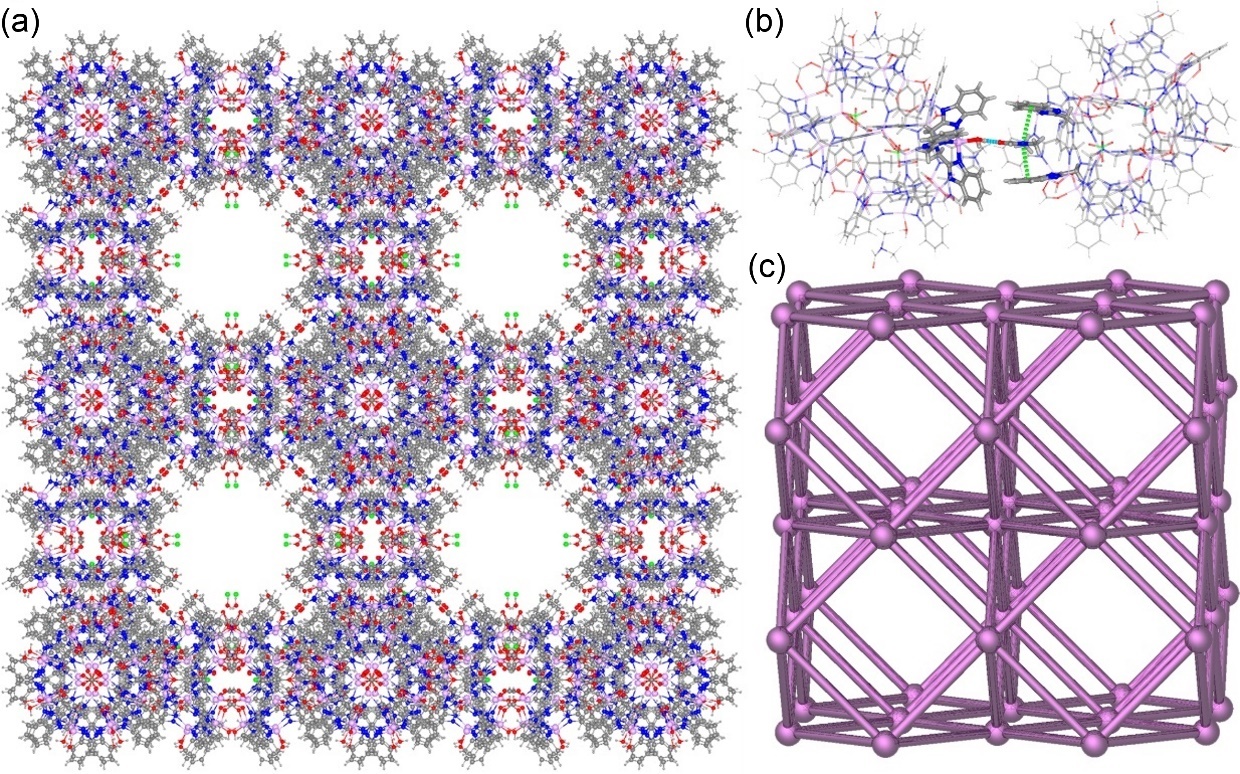


**Figure S6**. The connection between Zn_20_-MOC in the lattice of Zn_20_-MOC-SC. (a) The porous structure formed by the packing of **Zn_20_-MOC**. (b) The synergy of hydrogen bonding and amide‒*π* interactions between neighboring **Zn_20_-MOC**. (c) The ***reo*** net formed by **Zn_20_-MOC**.


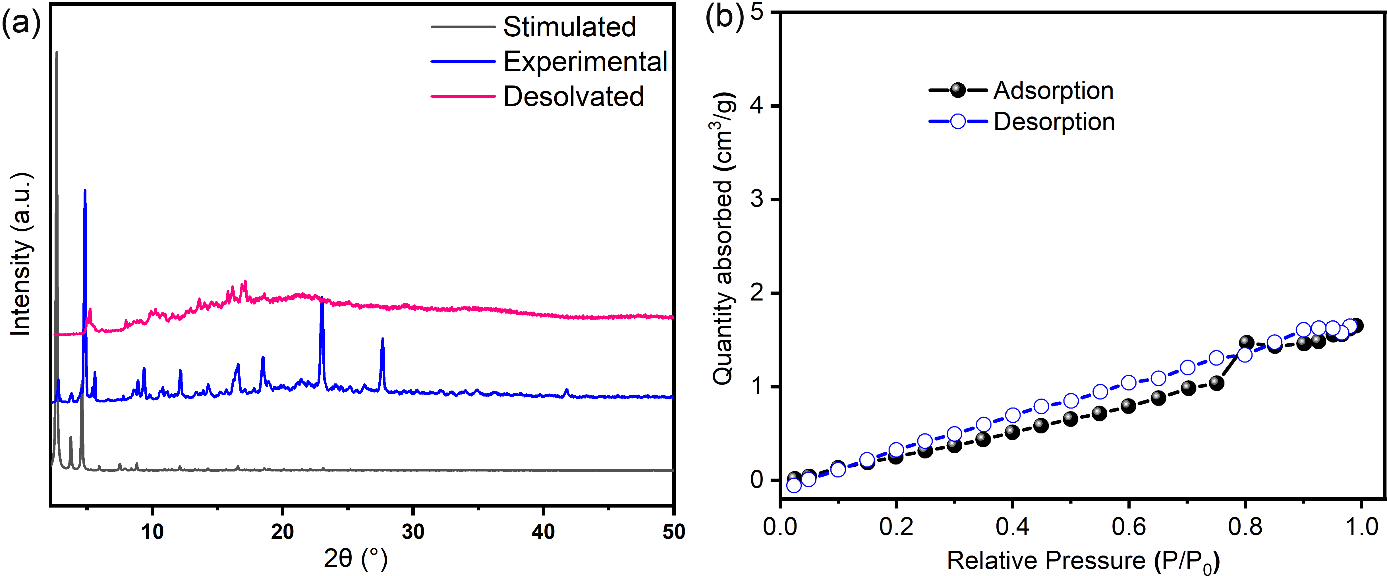


**Figure S7**. (a) Powder XRD patterns for **Zn_20_-MOC-SC**, (b) N_2_ sorption isotherms for desolvated **Zn_20_-MOC-SC** at 77 K**.**

**S3. Trimerization of 1,2-Diaminocyclohexane Catalyzed by Zn_20_-MOC**


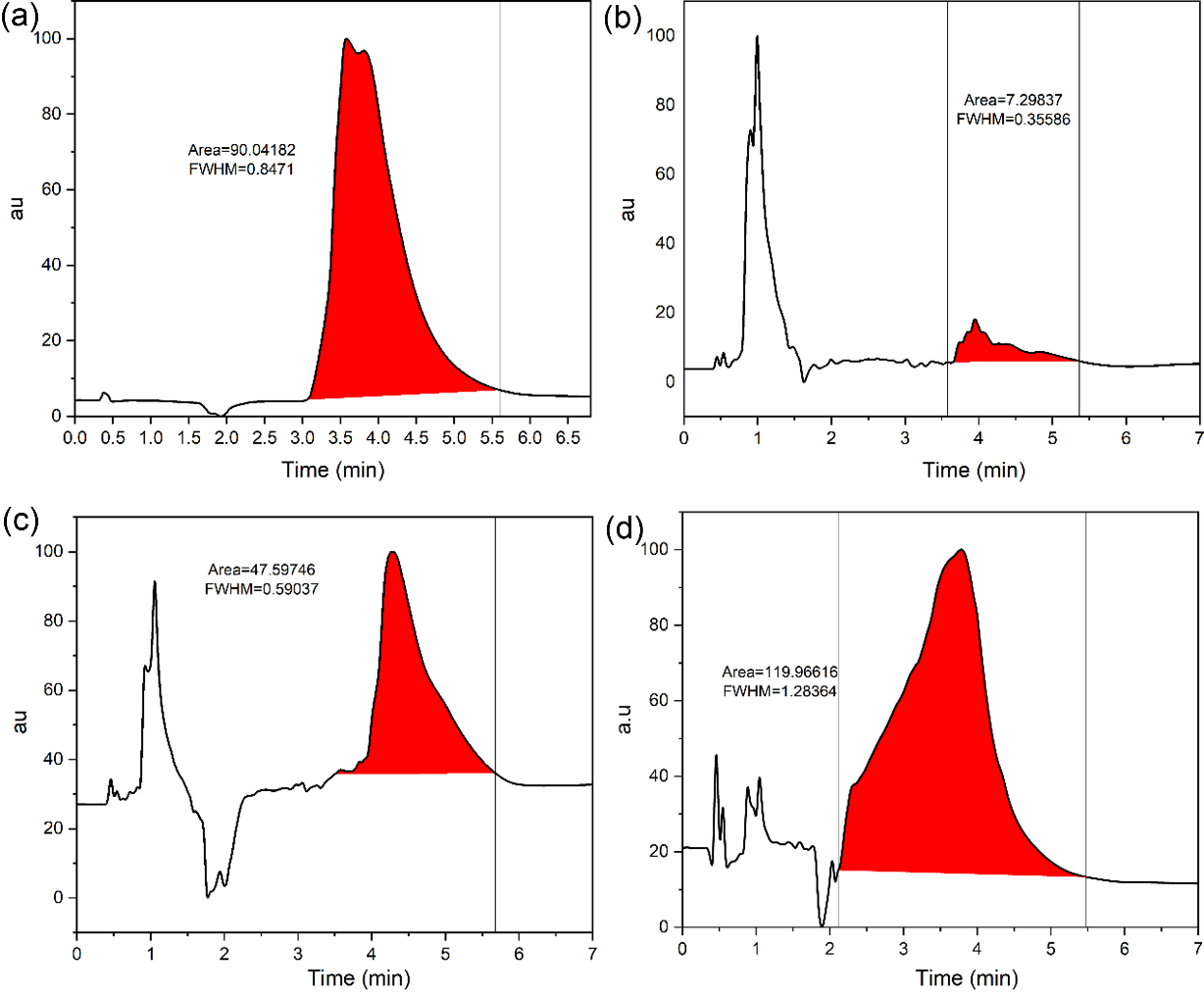


**Figure S8**. HPLC spectra. HPLC spectrum for standard sample (a), Entry 7 (b), Entry 10 (c), and Entry 1 (d).


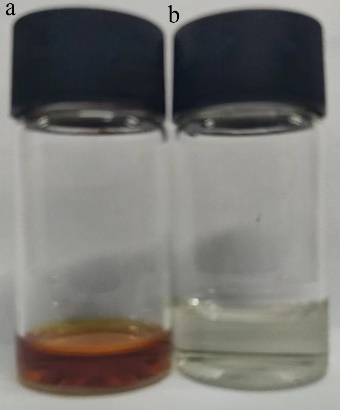


**Figure S9**. Detection of NH_4_^+^. The reactant solution after the 1,2-diaminocyclohexane trimerization after (a) and before (b) adding Nessler's reagent.


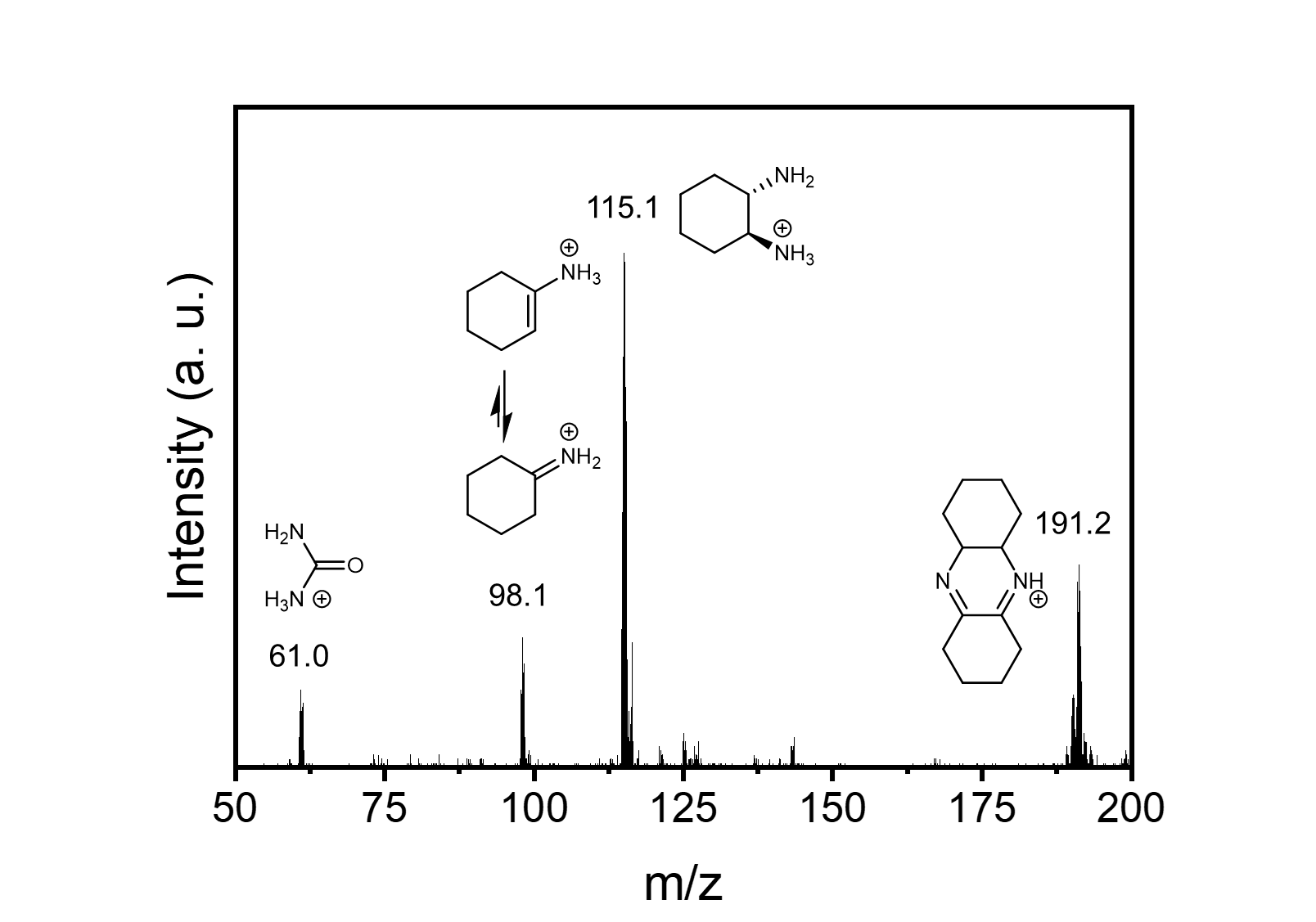


**Figure S10**. ESI-MS spectrum for the solution after the trimerization with **Zn_20_-MOC** as the catalyst showing urea as a byproduct.


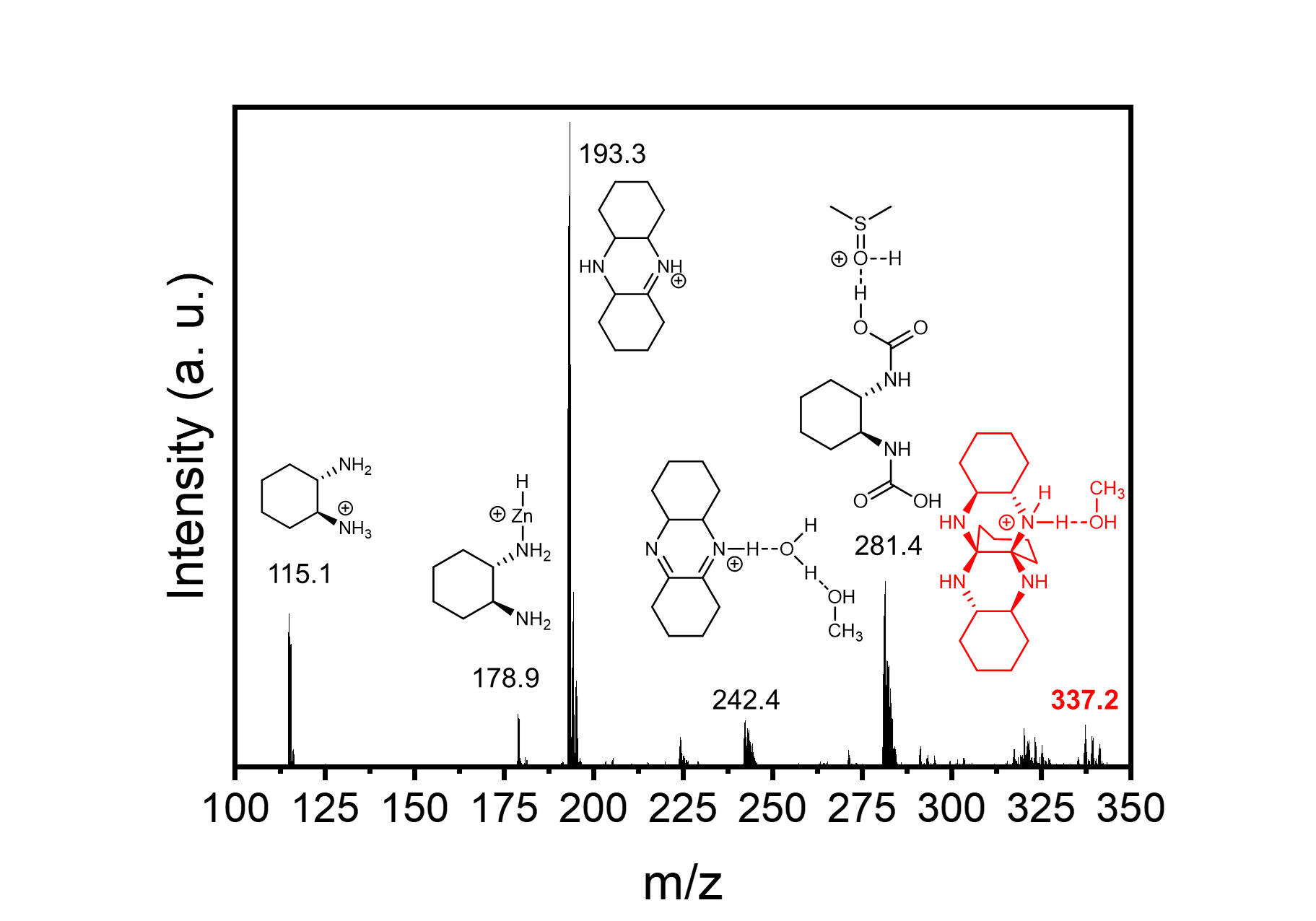


**Figure S11**. ESI-MS spectrum for the reactant solution with DMSO as the solvent and **Zn_20_-MOC** as the catalyst in air (Entry. 1).


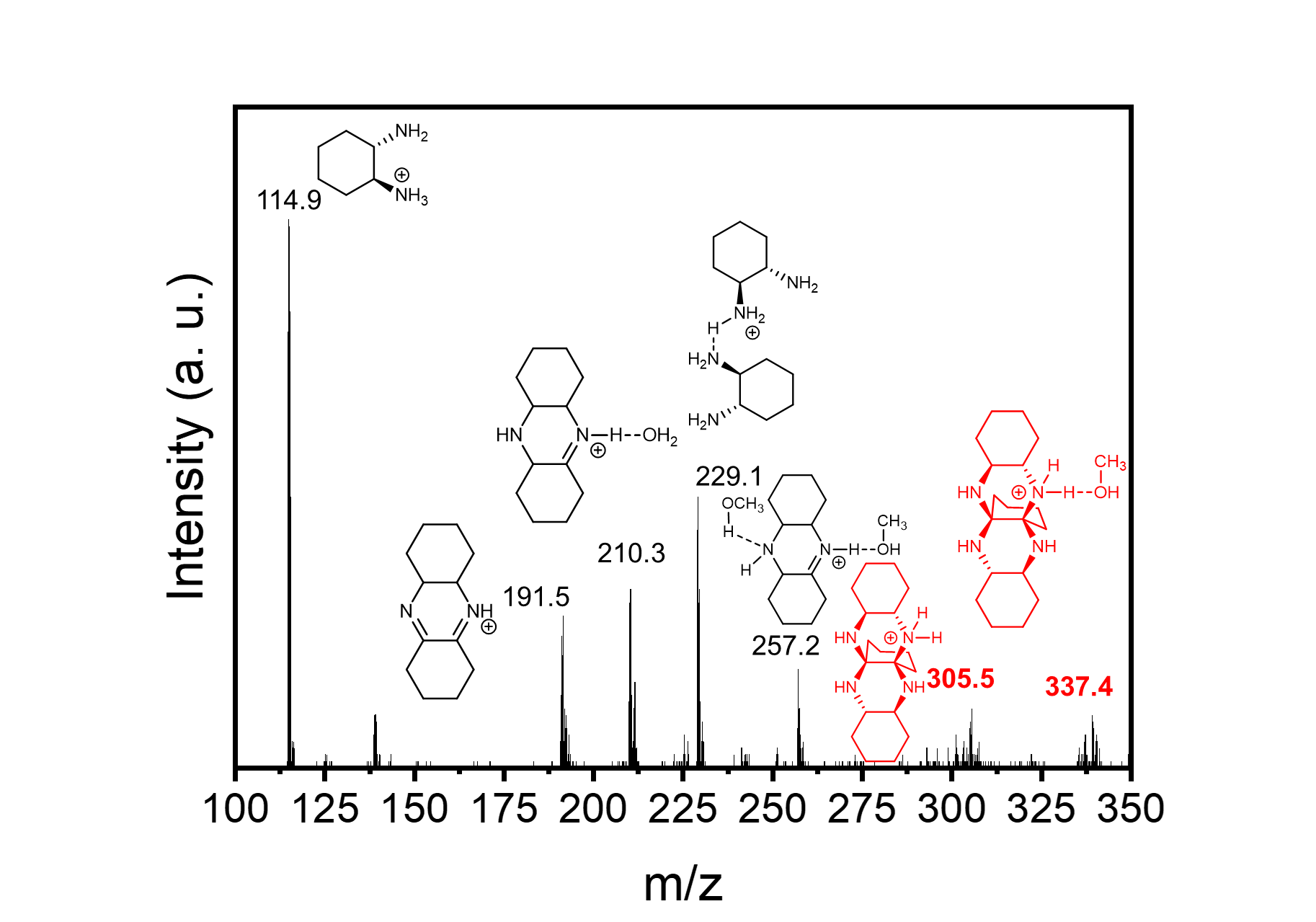


**Figure S12**. ESI-MS spectrum for the reactant solution with MeOH as the solvent and **Zn_20_-MOC** as the catalyst in air (Entry. 2).


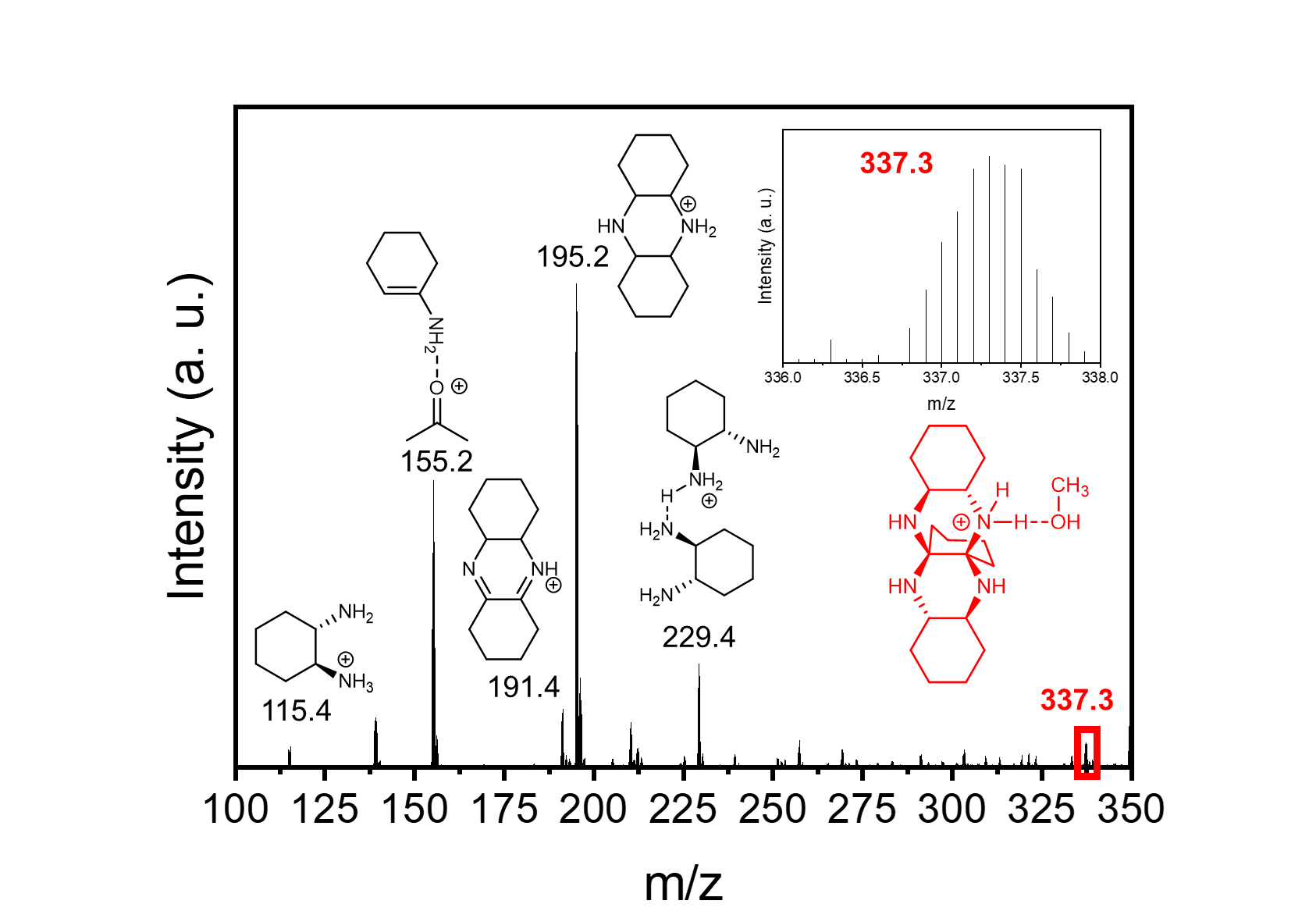


**Figure S13**. ESI-MS spectrum for the reactant solution with acetone as the solvent and **Zn_20_-MOC** as the catalyst in air (Entry. 3).


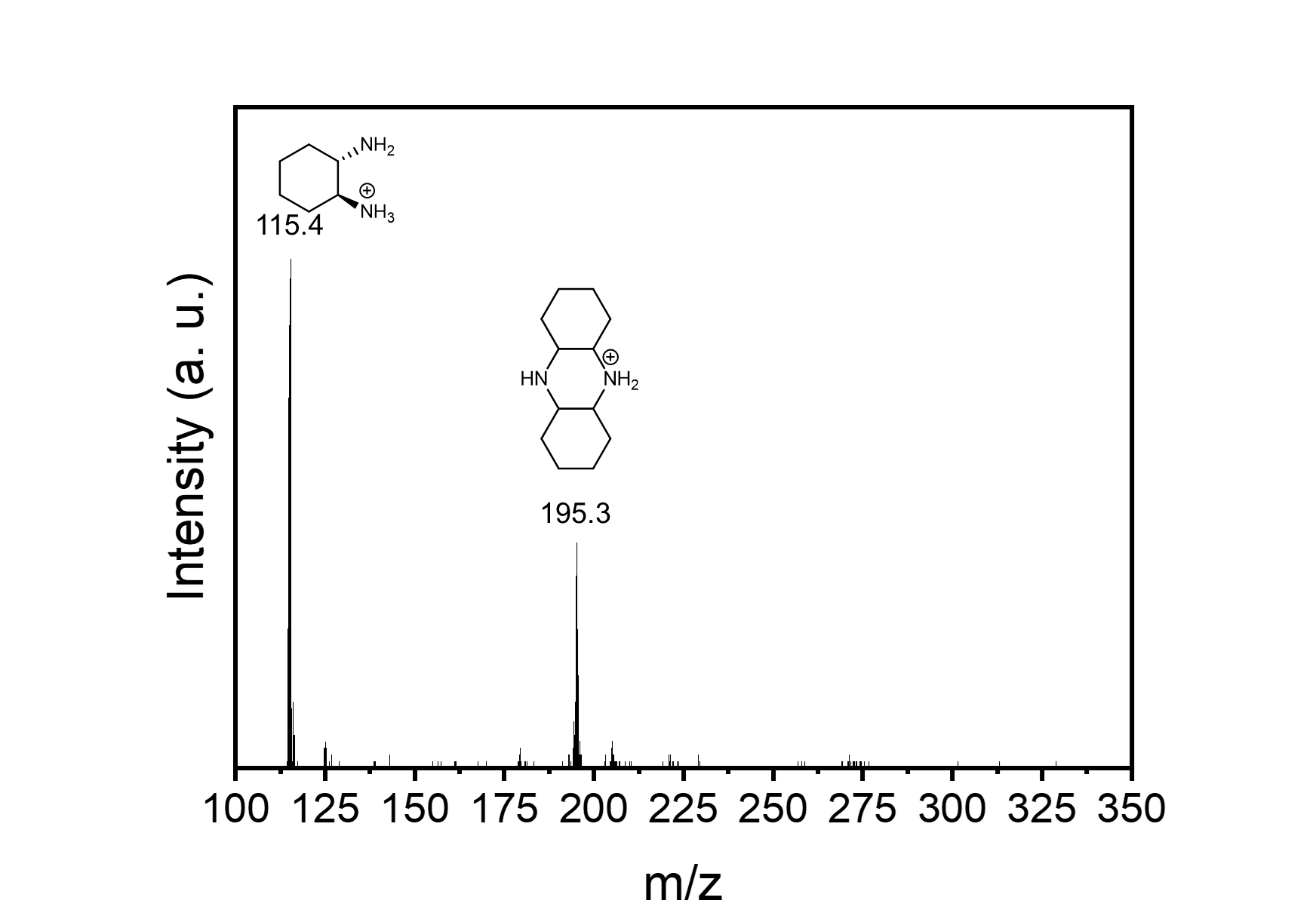


**Figure S14**. ESI-MS spectrum for the reactant solution with DMSO as the solvent and **Zn_20_-MOC** as the catalyst in N_2_ (Entry. 4).

^
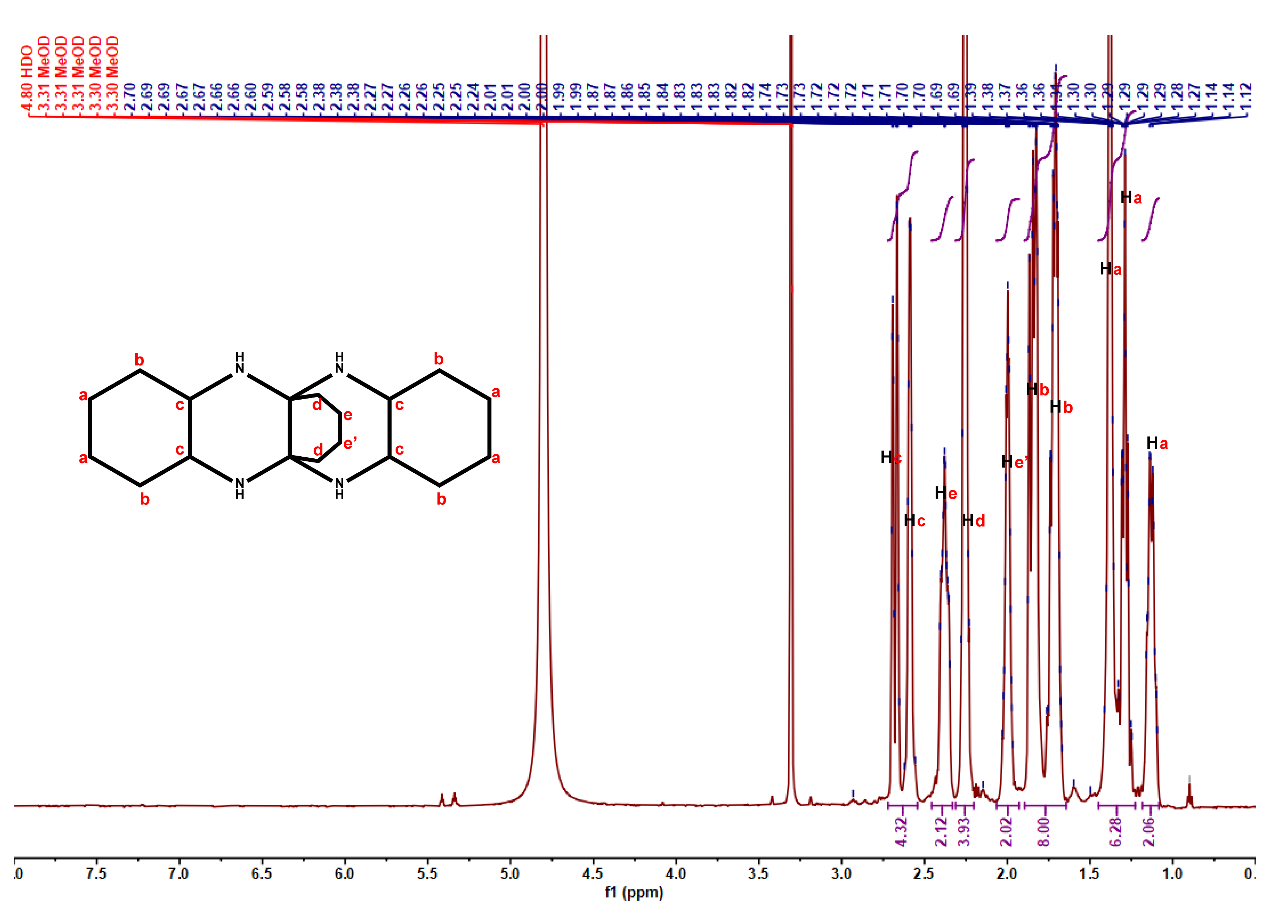
^

**Figure S15**. ^1^H NMR spectrum for **2**. ^1^H NMR (600 MHz, MeOD) δ 2.72 – 2.54 (m, 4H), 2.46 – 2.33 (m, 2H), 2.26 (dq, *J* = 8.6, 2.7 Hz, 4H), 1.99 (dtt, *J* = 7.9, 5.3, 2.6 Hz, 2H), 1.90 – 1.64 (m, 8H), 1.45 – 1.23 (m, 6H), 1.18 – 1.08 (m, 2H).


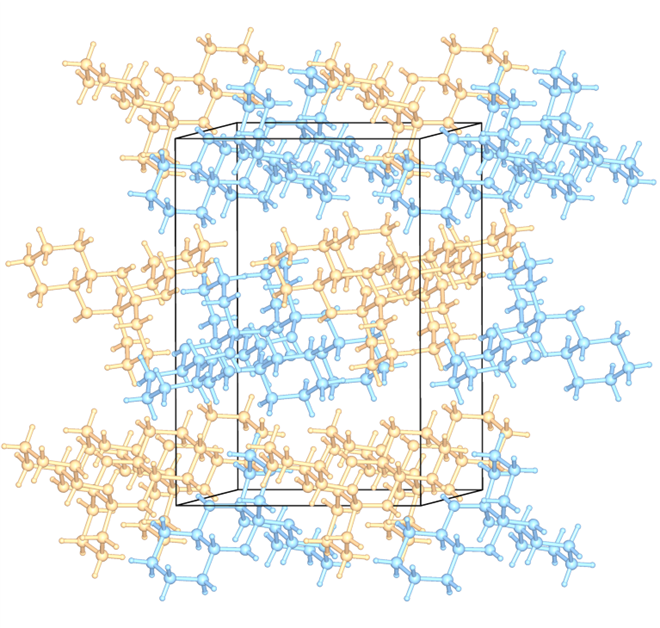


**Figure S16**. The packing of both the enantiomers of complex **2** in lattice.


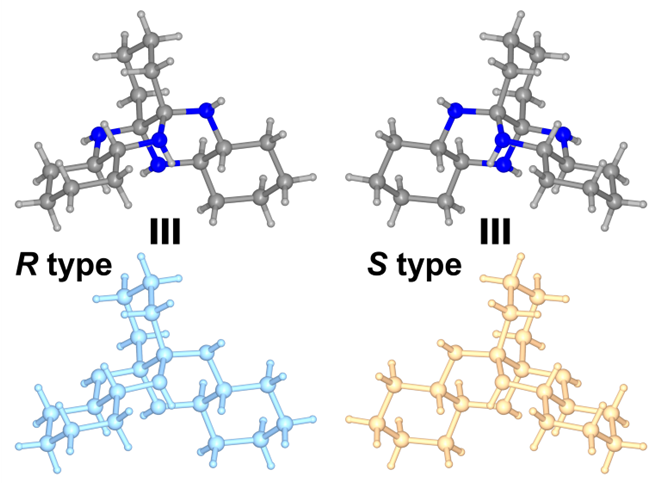


**Figure S17**.The structures for both the enantiomers of complex **2**.


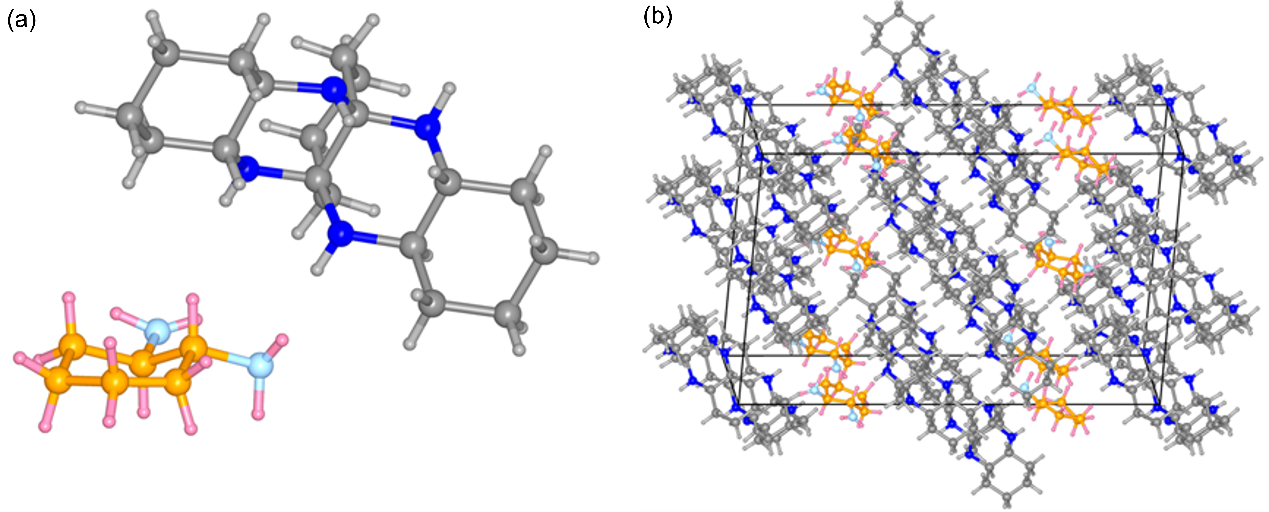


**Figure S18**. Crystal structure for **2_2_·1**. (a) The asymmetric unit for **2_2_·1**. (b) Packing of **1** and **2** in the lattice of **2_2_·1**.

**S4. Identification of the Enzyme-Like Catalytic Sites in Zn_20_-MOC**


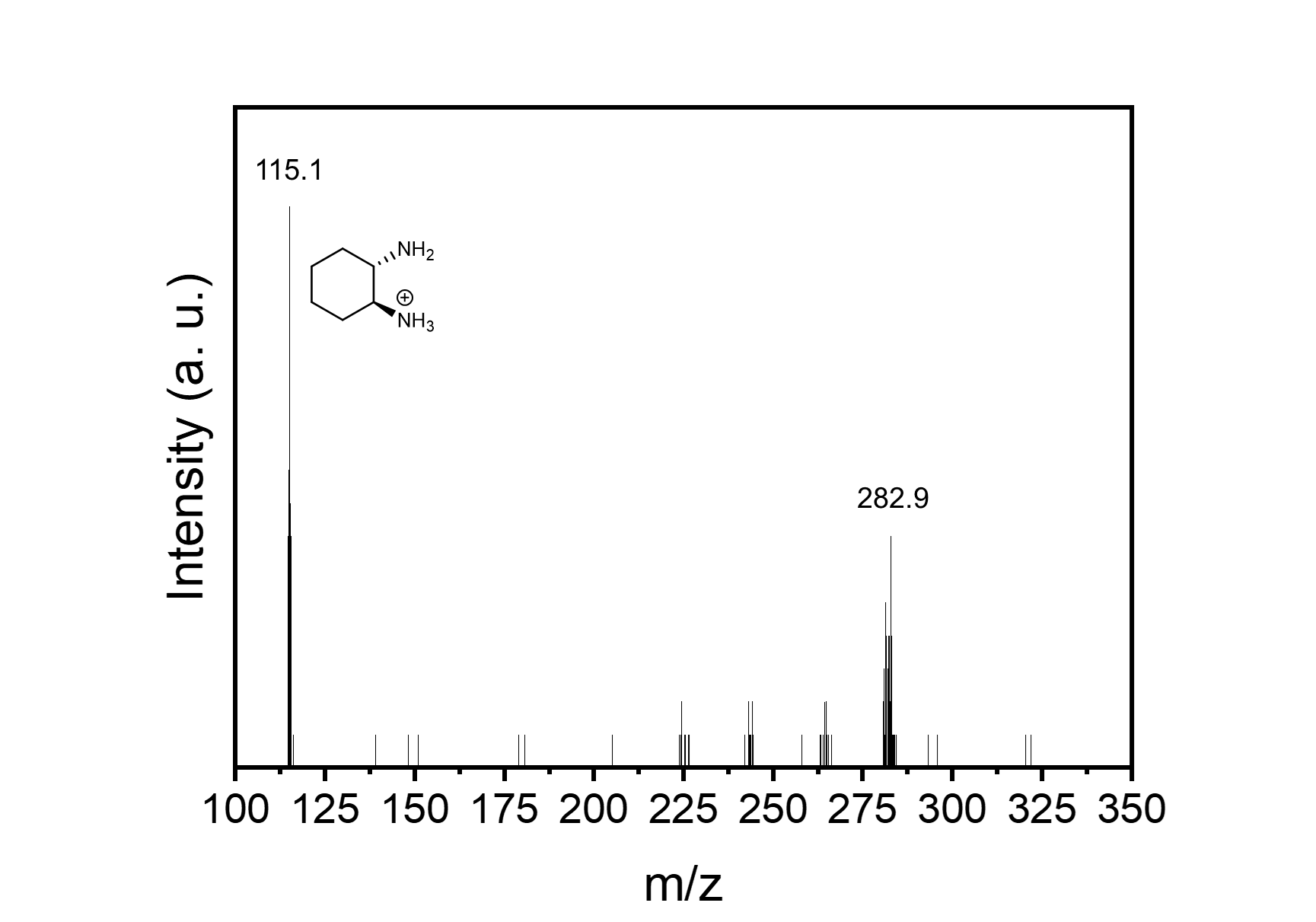


**Figure S19**. ESI-MS spectrum for the reactant solution with Zn(ClO_4_)_2_ as the catalyst (Entry. 5).


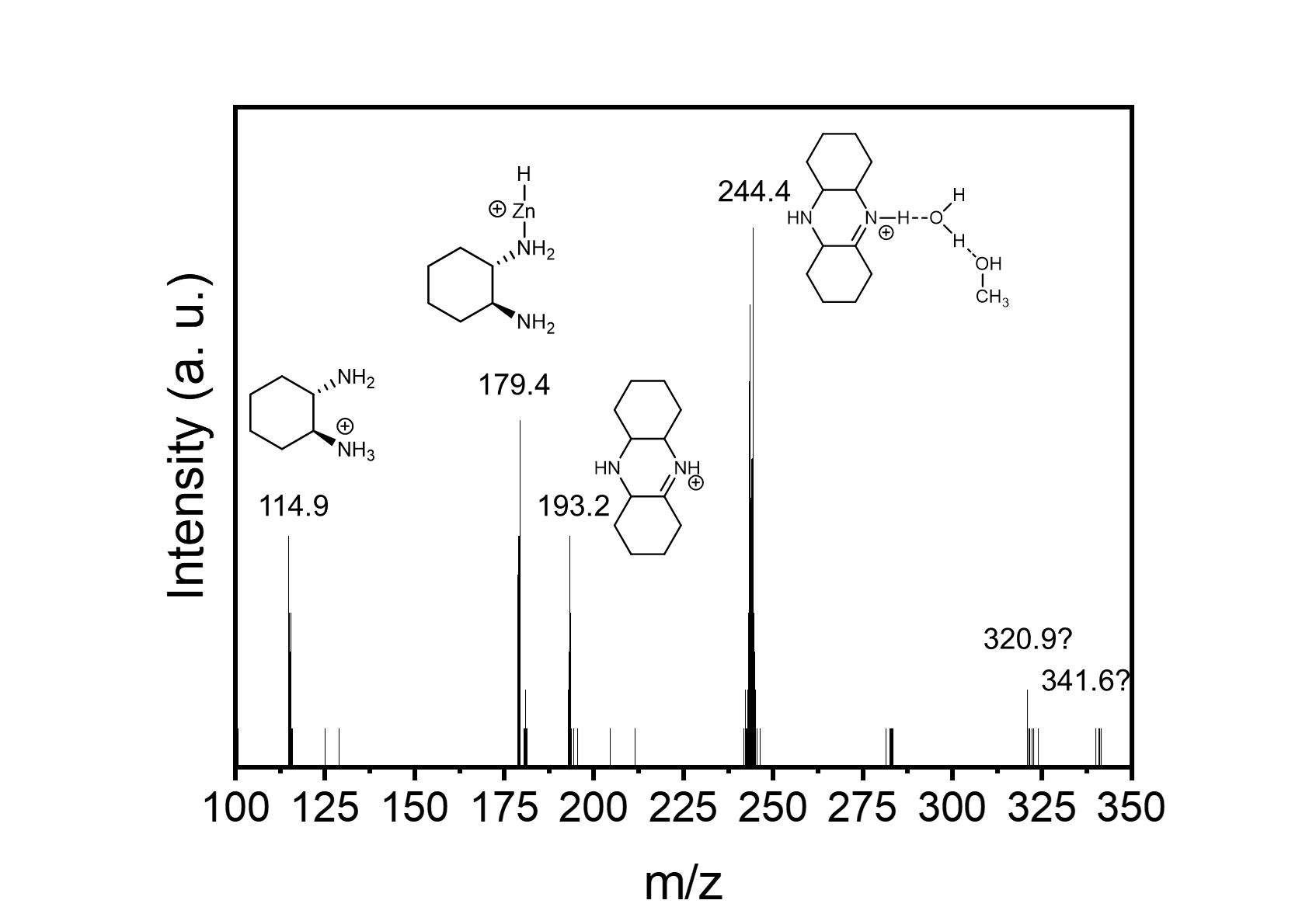


**Figure S20**. ESI-MS spectrum for the reactant solution with the mixture of Zn(ClO_4_)_2_ and H_3_L as the catalyst (Entry. 6).


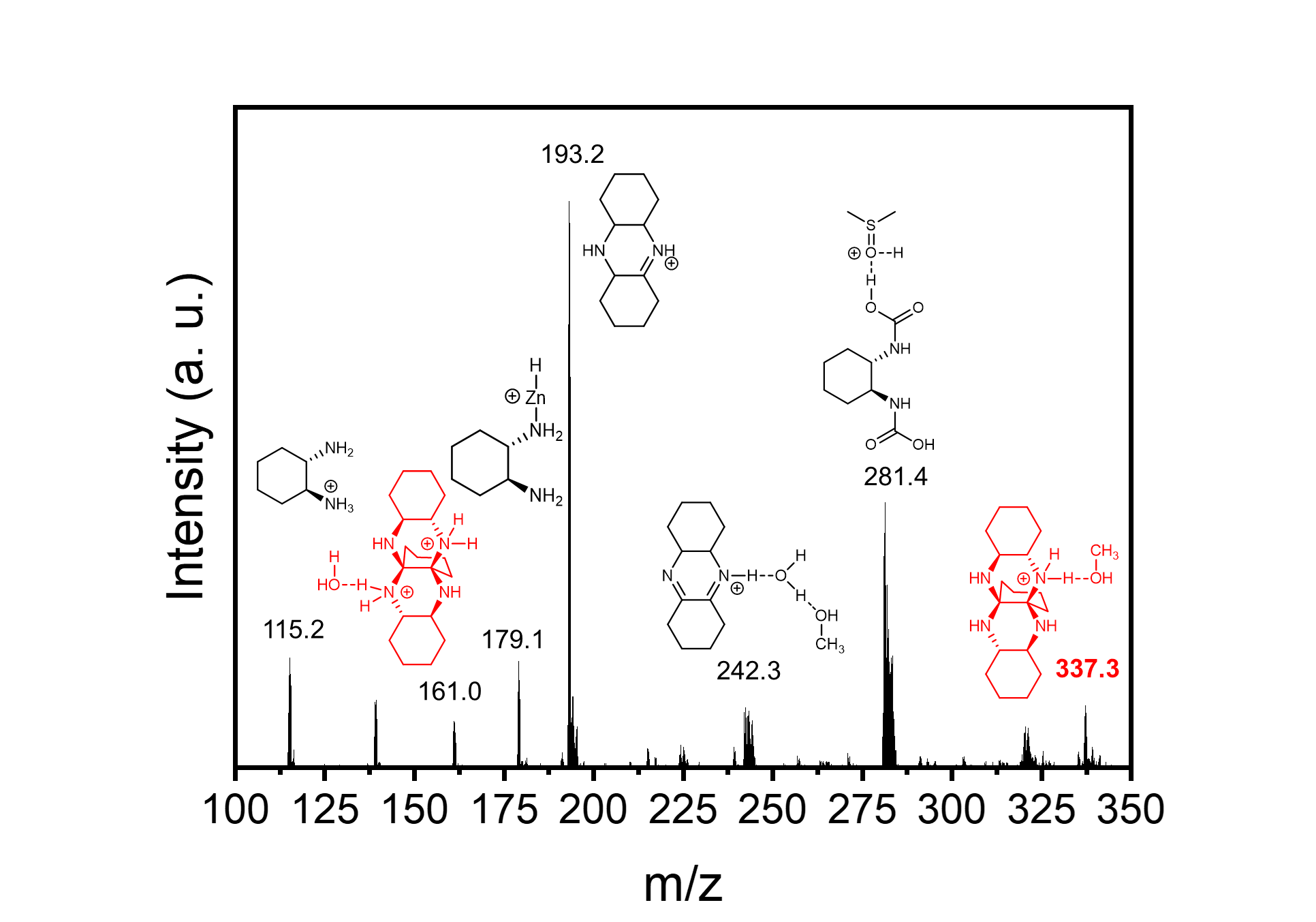


**Figure S21**. ESI-MS spectrum for the reactant solution with the mixture of Zn(HCOO)_2_ and H_3_L as the catalyst (Entry. 7).


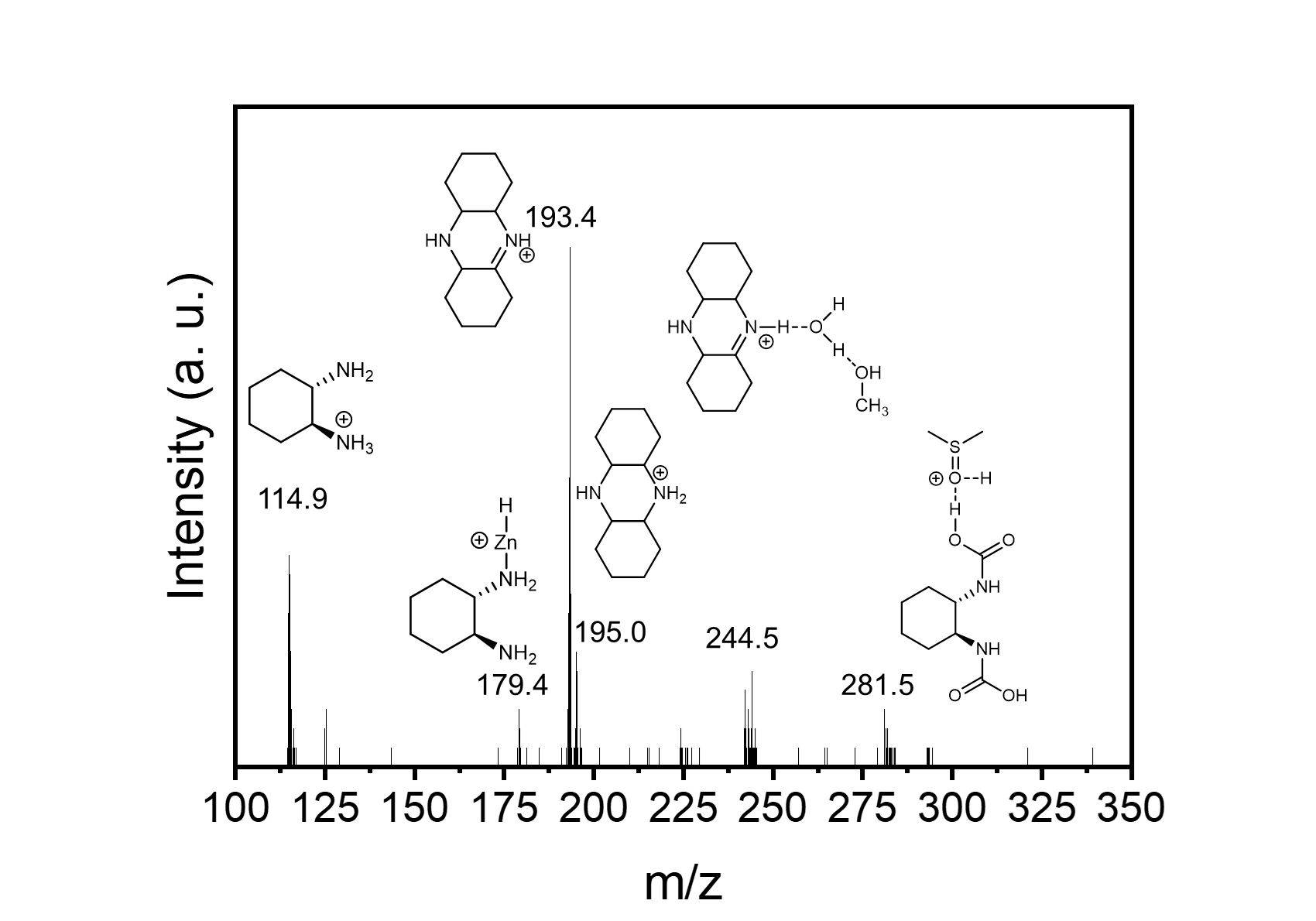


**Figure S22**. ESI-MS spectrum for the reactant solution with the mixture of ZnCl_2_ and H_3_L as the catalyst (Entry. 8).


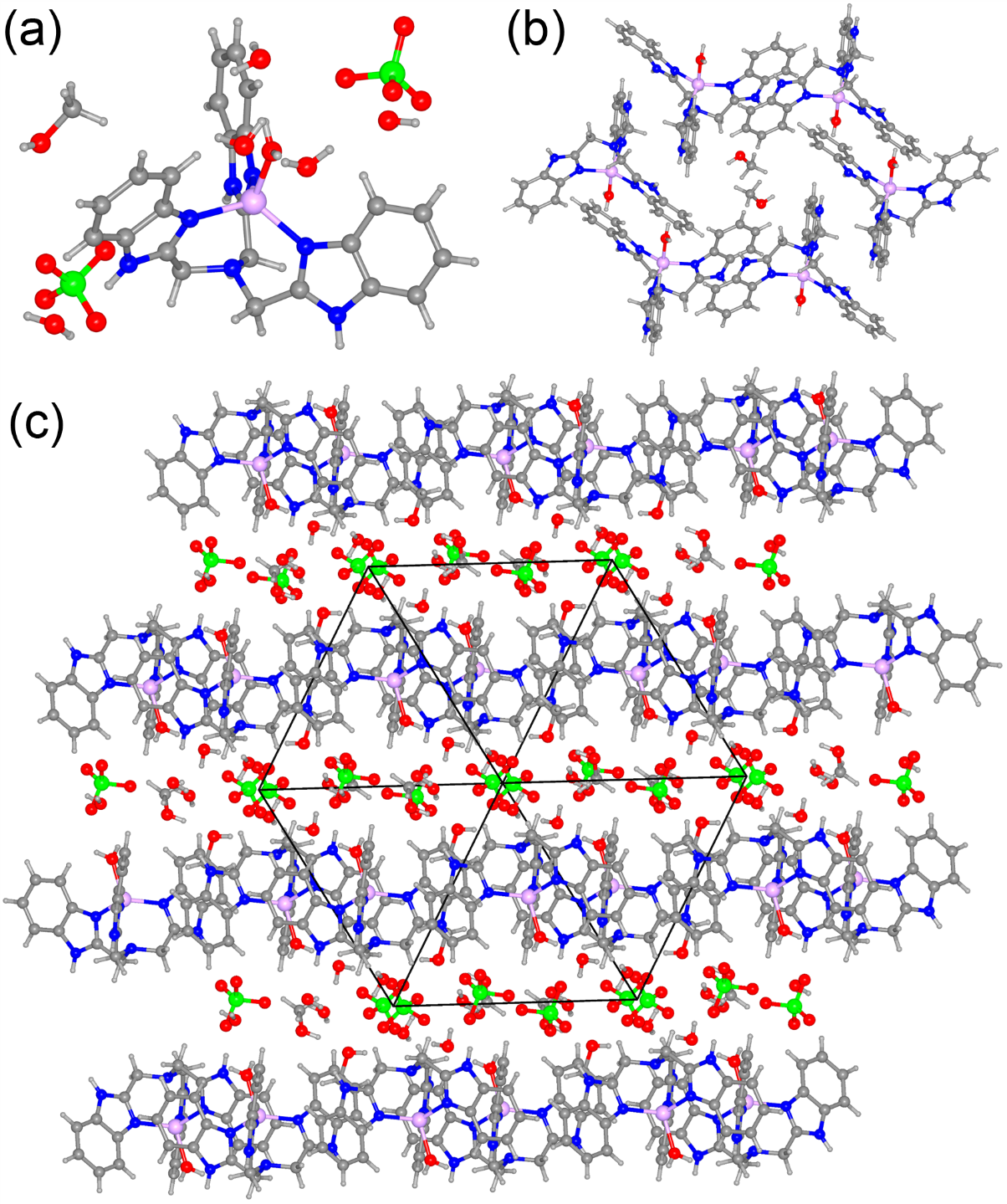


**Figure S23**. Crystal structure for **Zn_1_**. **a** Asymmetric unit for **Zn_1_**. **b** The interaction between neighboring {Zn^II^(L)(H_2_O)} motifs in **Zn_1_**. **c** The packing of {Zn^II^(L)(H_2_O)} motifs in **Zn_1_**.


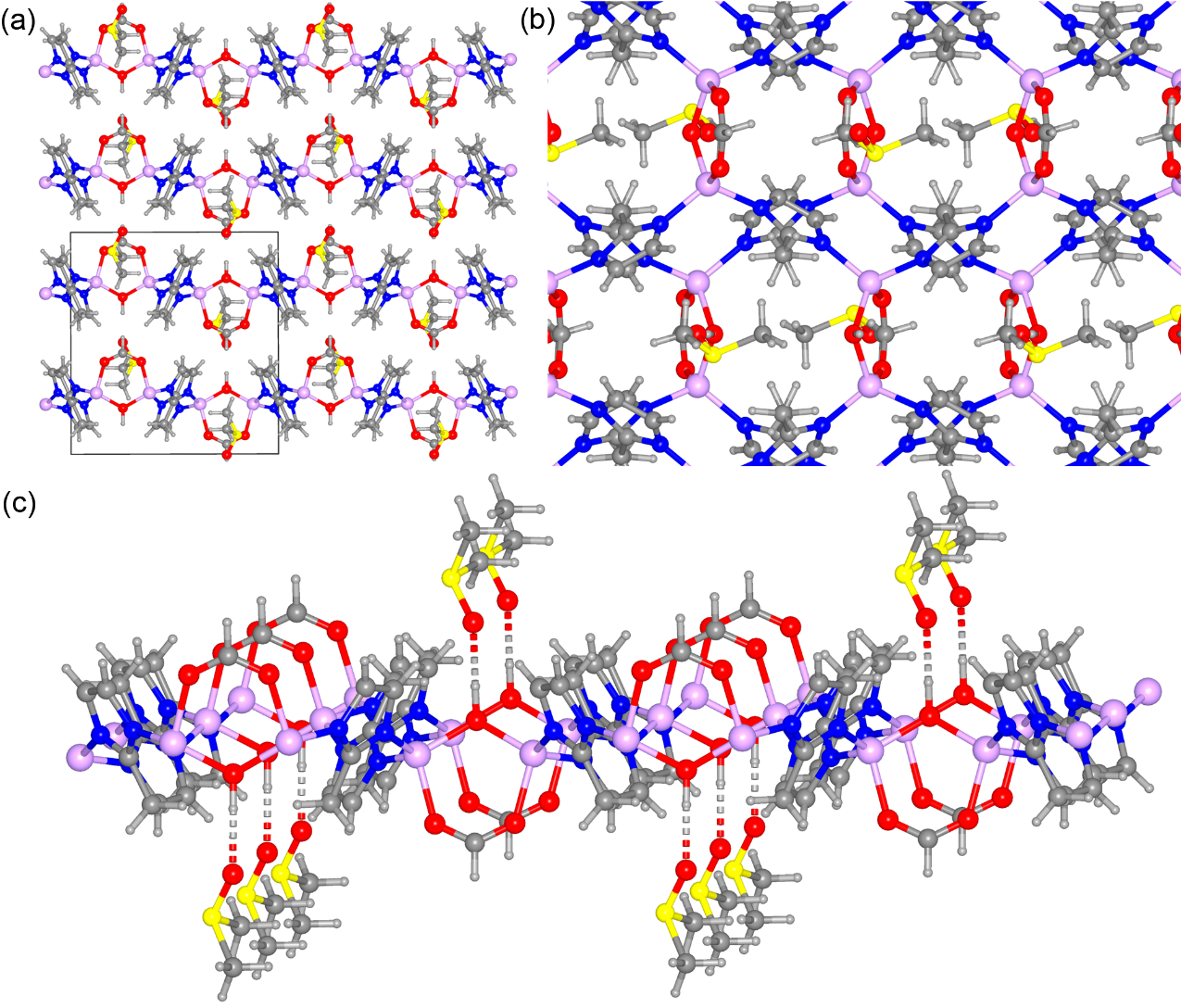


**Figure S24**. Crystal structure for **Zn_2_-MOF**. **a** Packing of the 2D layer structure in **Zn_2_-MOF**. **b** 2D layer structure in **Zn_2_-MOF**. **c** Hydrogen bonding between the 2D layer structure and DMSO in **Zn_2_-MOF**.


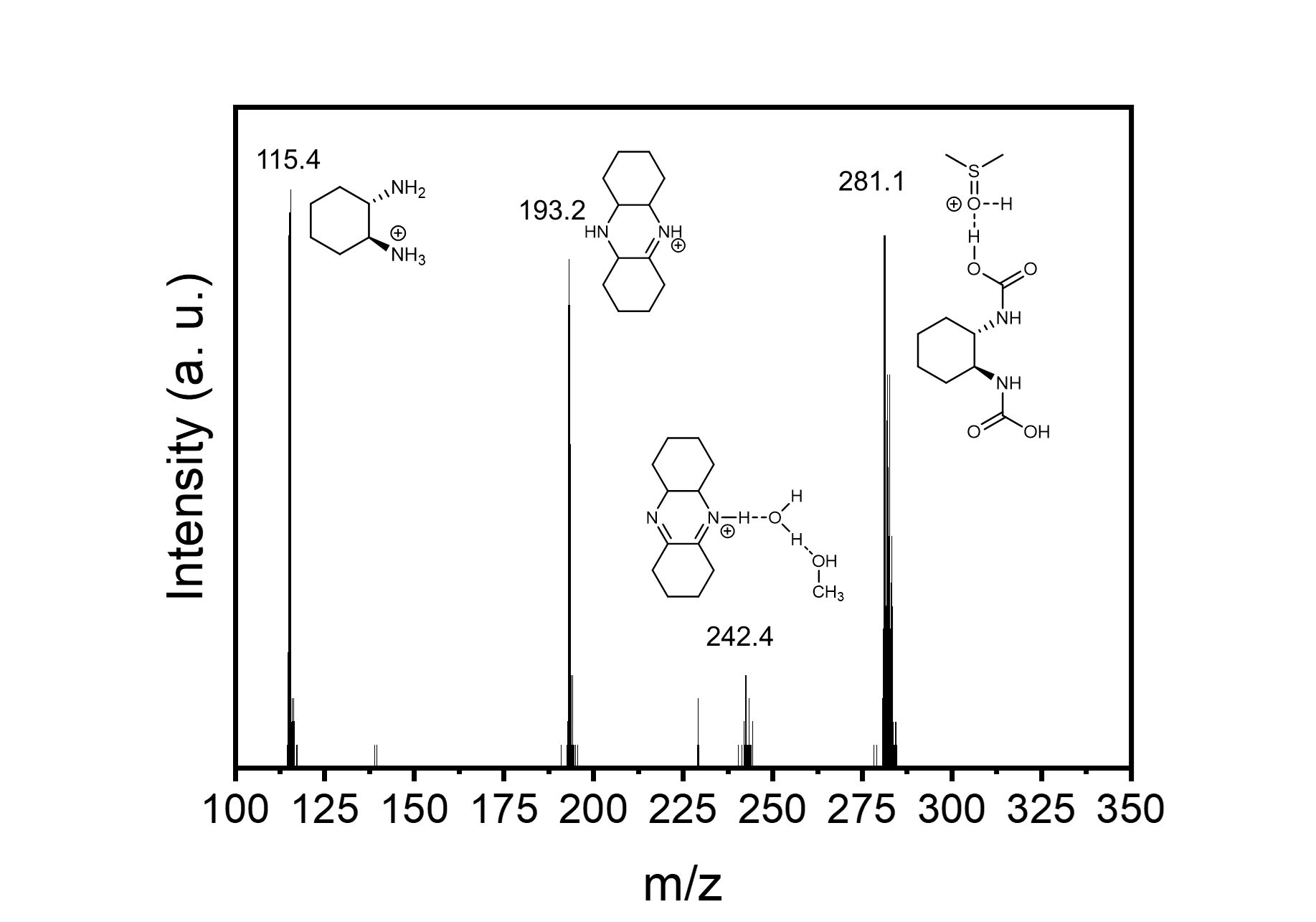


**Figure S25**. ESI-MS spectrum for the reactant solution with **Zn_1_** as the catalyst (Entry. 9).


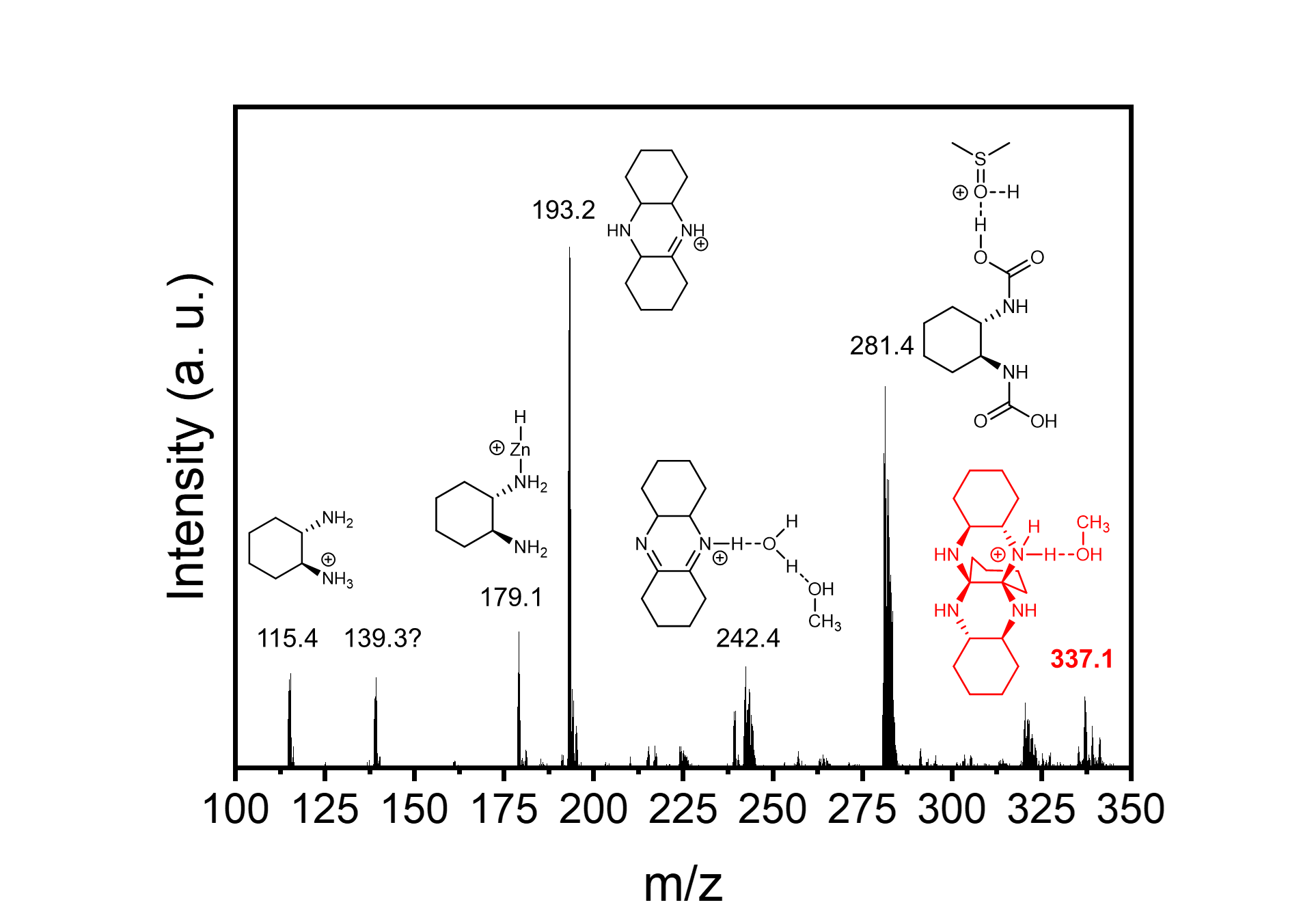


**Figure S26**. ESI-MS spectrum for the reactant solution with **Zn_2_-MOF** as the catalyst (Entry. 10).

**S5. Proposed Catalytic Mechanism**


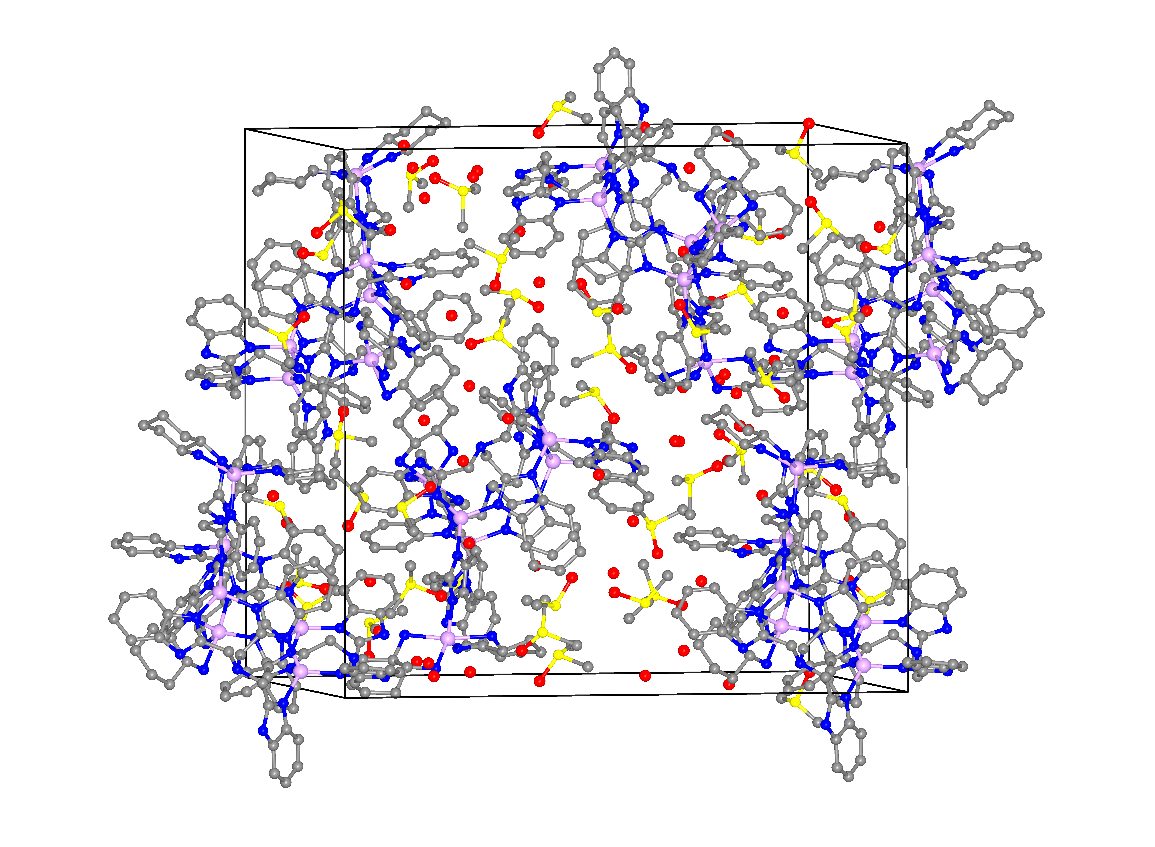


**Figure S27**. The packing of Zn_6_(L)_4_(**1**)_4_ clusters in **Zn_6_**.


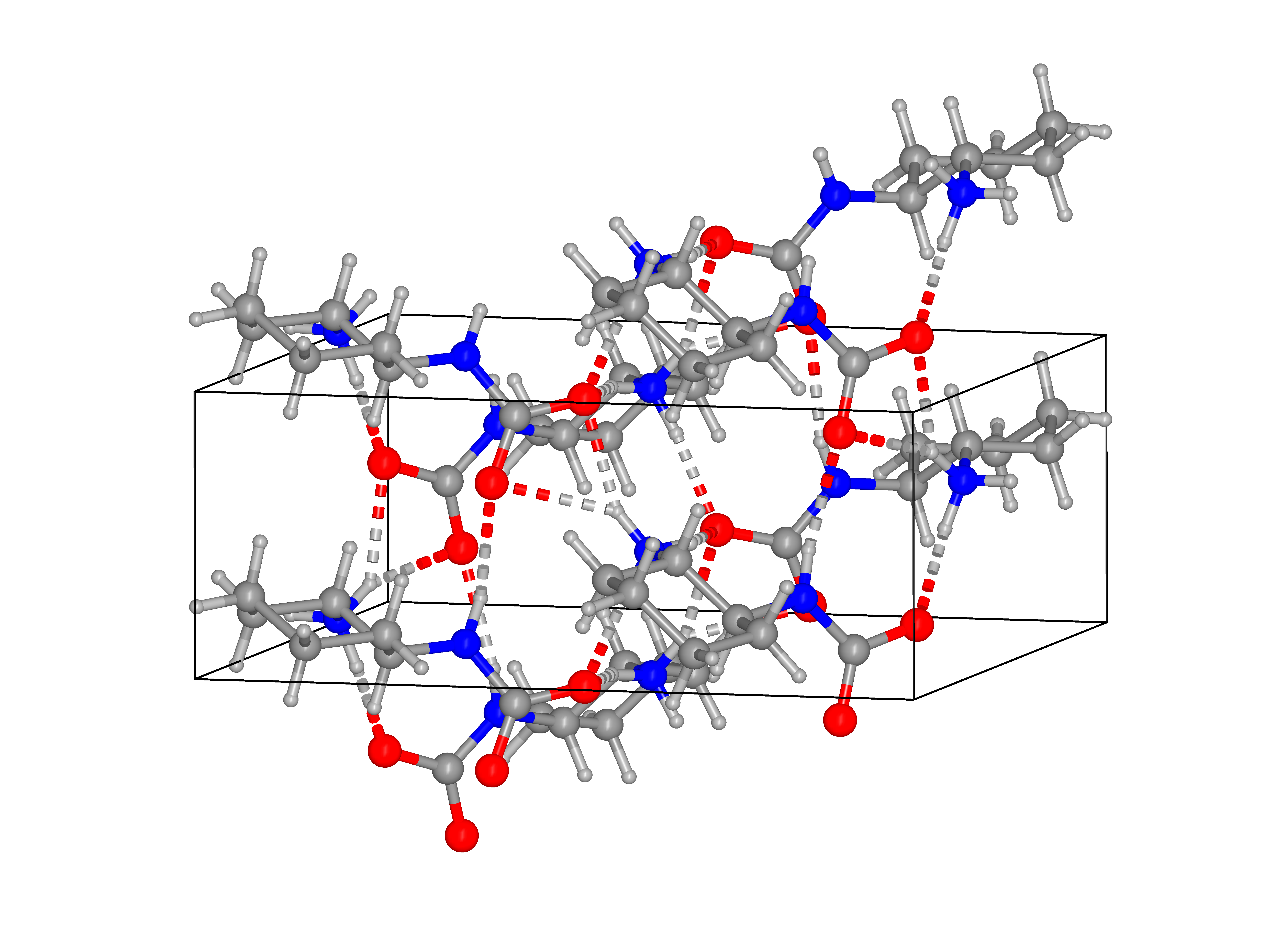


**Figure S28**. Crystal structure of (2-aminocyclohexyl)carbamic acid (**inter. 5**).





**Scheme S1**. Proposed pathways for dimerization of **1** catalyzed by the dinuclear site.
